# Mathematical modeling for a primitive form of habituation in an amoeba

**DOI:** 10.1007/s00285-026-02438-7

**Published:** 2026-08-17

**Authors:** Kota Nishi, Atsushi Tero, Yukinori Nishigami, Toshiyuki Nakagaki

**Affiliations:** 1 Joint Graduate School of Mathematics for Innovation, Kyushu University, Fukuoka, Japan; 2 Institute of Mathematics for Industry, Kyushu University, Fukuoka, Japan; 3 Research Institute for Electronic Science, Hokkaido University, Hokkaido, Japan

**Keywords:** 92-10, 37N25

## Abstract

Learning abilities, once thought to be unique to higher animals, have been reported to exist in their primitive form in single-celled organisms. This has triggered a growing interest in carefully examining the nature and mechanisms of the primitive versions of learning abilities, which would provide important clues for understanding the evolution of behavioral capabilities in organisms. In this study, we focused on previous experimental studies showing that the slime mold *Physarum polycephalum*, a model organism for studying protist behavior, exhibits the ability to adapt to chemical environments. We propose a possible dynamic mechanism underlying this habituation, reproducing reported experimental observations with accuracy. By refining a mathematical model that was as simple as possible and based on non-specific biochemical processes within cells, we clarified a plausible mechanistic framework. Based on these results, we examined the similarities and differences between this framework and previously proposed habituation models of single-cell movement and animal neural-circuit regulation. These findings are significant because they open new avenues for research into the generality and evolutionary origins of acclimation learning.

**Supplementary Information:**

The online version contains supplementary material available at https://doi.org/10.1007/s00285-026-02438-7.

## Introduction

Studies on information processing in simpler organisms, such as single-celled protists that can evoke an adaptive response to complex environmental situations, have deepened our understanding of cognitive behavior in neural organisms (Lyon et al. 2021). Several types of adaptive behavior have been reported in the amoeboid organism *Physarum polycephalum*, making it an important model organism for elucidating a primitive form of cognitive function in higher animals (Reid 2023).

Plasmodium of *P. polycephalum* is an amoeba-like multinucleated unicellular organism with a large sheet-like body up to several $\hbox {m}^2$. Its protoplasm exists in two phases; protoplasmic gel (ectoplasm), which exhibits passive elasticity and active rhythmic contractility, and protoplasmic sol (endoplasm), which is a viscous liquid. Inside the sheet-like body of plasmodium, an intricate network of veins (tubular channels for protoplasmic streaming) develops toward the rear from the extending front.

The ectoplasmic gel periodically contracts based on the biochemical regulation of the actomyosin system, and these self-sustained oscillations of contraction show some phase and force differences within the body. As this contraction generates pressure on the sol, these differences cause it to flow within the body through the tube network. This sol flow causes amoeboid migration of plasmodium while inducing major morphological changes in the tube network.

Plasmodium can solve difficult foraging problems by reforming its efficient tube-network (Nakagaki et al. 2000; Tero et al. 2010), and can memorize and anticipate the environmental periodic changes as well (Saigusa et al. 2008). These adaptive behaviors and their underlying mechanisms have been analyzed using mathematical modeling (Nakagaki et al. 2007; Saigusa et al. 2008; Tero et al. 2007, 2010).

As this mathematical model elucidated the key mechanism underlying adaptive behavior, it was applicable to similar types of behavior in higher animals, such as the social design of public transportation networks in human society (Tero et al. 2010) and ant trail formation in complicated landscapes (Ma et al. 2013).

As discussed above, studies on *P. polycephalum* have provided a new perspective on the evolution of behavioral intelligence (right from cells to animal societies). Along this line of study, we aimed to investigate habituation in *P. polycephalum* and analyze the underlying mechanism by mathematical modeling.

Habituation, a type of learning, refers to the decrease in behavioral responses when stimuli are repeatedly presented (Rankin et al. 2009). Habituation is distinct from sensory adaptation, sensory fatigue, and motor fatigue (Rankin et al. 2009), and is characterized by ten hallmarks (Table 1). Several studies have attempted to elucidate the mechanisms underlying habituation. Most habituation studies have focused on multicellular organisms that possess a nervous system (Castellucci et al. 1970; Johnson and Wuensch 1994).

**Table 1:** The hallmarks of habituation

#1	$\text {habituation}^{1}$	Repeated application of a stimulus results in a progressive decrease in some parameter of a response to an asymptotic level. This change may include decreases in frequency and/or magnitude of the response.
#2	spontaneous $\text {recovery}^{1}$	If the stimulus is withheld after response decrement, the response recovers at least partially over the observation time.
#3	potentiation of $\text {habituation}^{1}$	After multiple series of stimulus repetitions and spontaneous recoveries, the response decrement becomes successively more rapid and/or more pronounced
#4	frequency $\text {sensitivity}^{2}$	Other things being equal, more frequent stimulation results in more rapid and/or more pronounced response decrement, and more rapid spontaneous recovery (if the decrement has reached asymptotic levels)
#5	intensity $\text {sensitivity}^{2}$	Within a stimulus modality, the less intense the stimulus, the more rapid and/or more pronounced the behavioral response decrement. Very intense stimuli may yield no significant observable response decrement.
#6	subliminal $\text {accumulation}^{2}$	The effects of repeated stimulation may continue to accumulate even after the response has reached an asymptotic level (which may or may not be zero, or no response). This effect of stimulation beyond asymptotic levels can alter subsequent behavior, for example, by delaying the onset of spontaneous recovery.
#7	stimulus $\text {specificity}^{2}$	Within the same stimulus modality, the response decrement shows some stimulus specificity.
#8	$\text {dishabituation}^{1}$	Presentation of a different stimulus results in an increase of the decremented response to the original stimulus.
#9	habituation of $\text {dishabituation}^{1}$	Upon repeated application of the dishabituating stimulus, the amount of dishabituation produced decreases.
#10	long-term $\text {habituation}^{1}$	Some stimulus repetition protocols may result in properties of the response decrement (e.g. more rapid rehabituation than baseline, smaller initial responses than baseline, smaller mean responses than baseline, less frequent responses than baseline) that last hours, days or weeks.

Feature descriptions are quoted from Rankin et al. (2009). ^1^ Names are adopted from Rankin et al. (2009). ^2^ Names are adopted from Eckert et al. (2024)

However, some single-celled microorganisms, such as ciliates (Wood 1988a, b; Rajan et al. 2023) and amoebae (Boisseau et al. 2016), also exhibit habituation. Habituation to mechanical stimuli has been extensively demonstrated in ciliates (Dussutour 2021). For example, in the ciliate *Stentor*, the contraction response to mechanical stimulation is an example of habituation (Wood 1988a). In this habituation, changes in intracellular $\hbox {Ca}^{2+}$ concentration are crucial (Dussutour 2021).

In amoebae, habituation to chemical stimulation has been observed in plasmodium of *P. polycephalum*, as demonstrated by the hallmarks of habituation (Boisseau et al. 2016). However, the underlying mechanisms have not been adequately explored. Therefore, in this study, we propose a mathematical model to reproduce habituation in *P. polycephalum*. We compared this model with previous models and discussed the commonalities in the habituation mechanism.

## Nature of habituation in plasmodium and aim of mathematical modeling

**Fig. 1:**
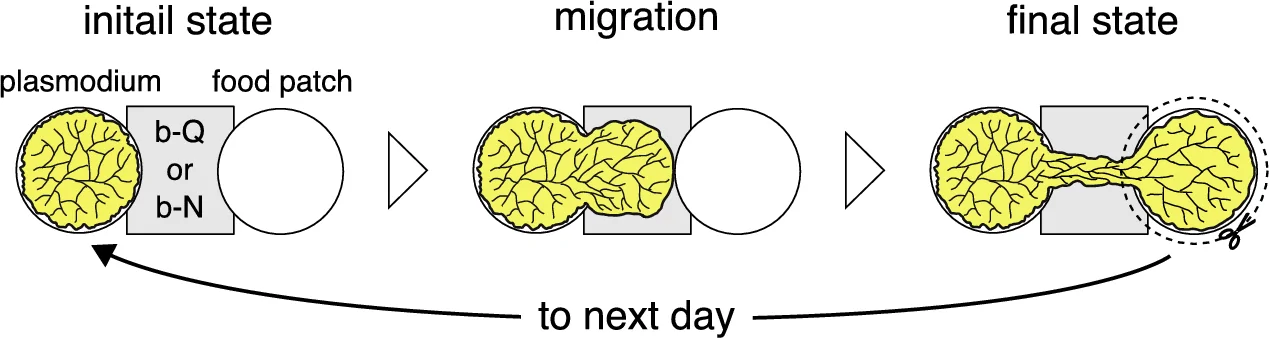
Illustration depicting the workflow of the bridge crossing experiment conducted by Boisseau et al. (2016). The b-Q represents the agar bridge containing quinine, and the b-N represents the normal agar bridge

**Fig. 2:**
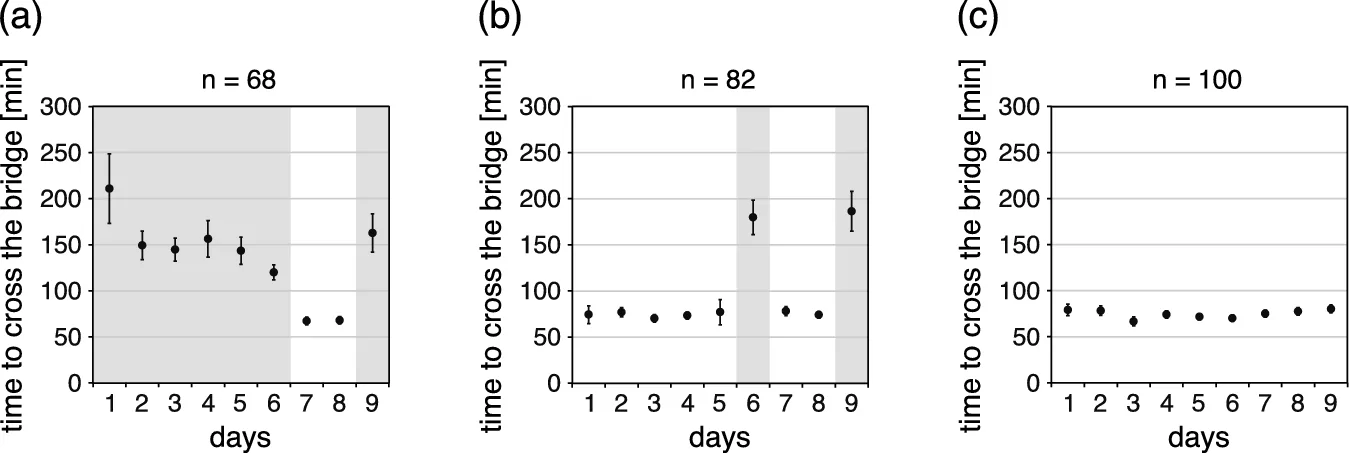
Time taken by plasmodium to cross the bridge from day 1 to 9 (Boisseau et al. 2016). Black point represents the mean time to cross the bridge, and the error bar represents the 95 percent confidence interval: the gray background represents the day when plasmodium crossed the b-Q, and the white background represents the day when plasmodium crossed the b-N. Sample sizes are n=68 in (a), n=82 in (b), and n=100 in (c). Data adapted from Boisseau et al. (2016)

Plasmodium usually locomotes when searching for food products. It decreases its speed when encountering quinine, a chemical repellent (Takagi et al. 2007). Boisseau et al. (2016) demonstrated the hallmarks of habituation in plasmodium by leveraging this property. They conducted bridge-crossing experiments on plasmodium (Fig. 1). In their experiment, a plasmodium was placed on a food patch, with a new food patch connected by one of two agar bridges: an agar bridge containing quinine (hereinafter, b-Q) or a normal agar bridge without quinine (hereinafter, b-N). The bridge was set between plasmodium and the new food patch, and plasmodium was then made to cross either the b-Q or the b-N once per day (Fig. 1). After the plasmodium moved to the food source, the corresponding patch was relocated to a new arena at the same time each day. Additionally, a new bridge and a fresh food source were introduced for the next trial (Fig. 1). They recorded the time from when the tip of plasmodium entered the bridge to when it reached a new food patch (hereinafter called the time to cross the bridge). From their experiments, the following observations were made: (i), the time to cross the b-Q decreased when plasmodium repeatedly crossed the b-Q (Fig. 2(a)), (ii), the time to cross the b-Q recovered after plasmodium crossed the b-N for 2 consecutive days (Fig. 2(a)), (iii), the time required to cross the b-N does not depend on the number of previous passes through the b-Q (Fig. 2 at day 7). In particular, features (i) and (ii) correspond to habituation and spontaneous recovery (hallmarks #1 and #2 in Table 1), respectively. Although feature (iii) is not directly related to habituation, feature (iii) suggests that plasmodium reflects the stimulus history of its motility only under necessary circumstances.

In this study, we developed a mathematical model to account for the three behavioral features (i) ∼ (iii). We formulated a locomotion model of plasmodium driven by a chemical reaction–diffusion process. In the next section, we describe how the physiological and behavioral properties of plasmodium are represented by a mathematical model.

## Formulation of the mathematical model

### Overview of our model

First, we present an overview of the formulated model. We developed the following model, which comprises the equation describing plasmodium locomotion (Eq. (1)) and the reaction–diffusion system regulating locomotion (Eqs. (2) and (3)).

1 $$\begin{aligned} \frac{d\ell }{dt}(t)&=\gamma (p_{\textrm{r}}(t)-p_{\textrm{f}}(t)), \end{aligned}$$

2 $$\begin{aligned} \frac{dp_{\textrm{f}}}{dt}(t)&= d(p_{\textrm{r}}-p_{\textrm{f}}) + F_{\textrm{f}}(p_{\textrm{f}}),\end{aligned}$$

3 $$\begin{aligned} \frac{dp_{\textrm{r}}}{dt}(t)&= r_{\textrm{v}}d(p_{\textrm{f}}-p_{\textrm{r}}) + F_{\textrm{r}}(p_{\textrm{r}}). \end{aligned}$$

where

4 $$\begin{aligned} F_{\textrm{f}}(p_{\textrm{f}})&= (1-h(t))(-a_{\textrm{de}}p_{\textrm{f}}+g_{\textrm{f}}) +h(t)(-a_{\textrm{de}}^{\textrm{s}}p_{\textrm{f}}+g_{\textrm{f}}^{\textrm{s}}), \end{aligned}$$

5 $$\begin{aligned} F_{\textrm{r}}(p_{\textrm{r}})&= -a_{\textrm{de}}p_{\textrm{r}}+g_{\textrm{r}}. \end{aligned}$$

**Fig. 3:**
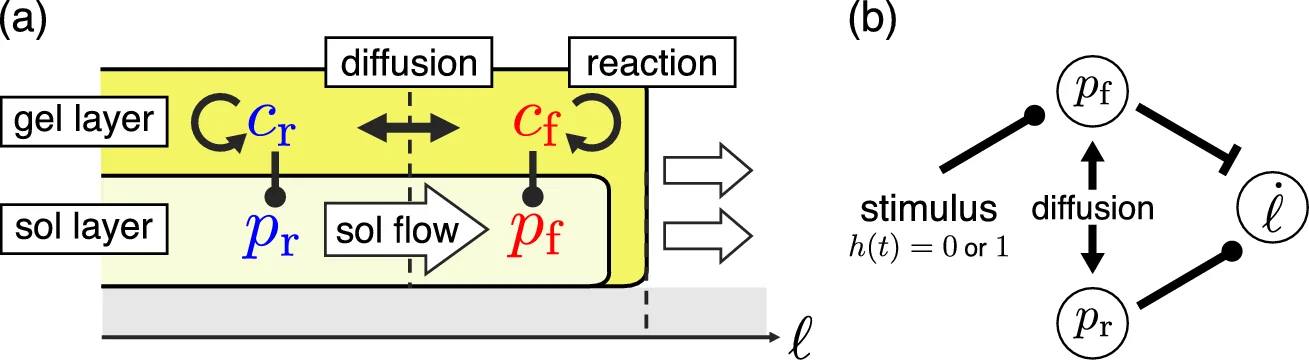
(a) The schematic illustration of a cross-section of plasmodium. Each of $c_{\textrm{f}}$ and $c_{\textrm{r}}$ represents a chemical that regulates the gel contraction. $p_{\textrm{f}}$ and $p_{\textrm{r}}$ represent the sol pressure. ℓ represents the coordinate of the tip position. A curved arrow represents a chemical reaction, a double-headed arrow represents the diffusion effect, and a line with a circle represents an activation effect. (b) The schematic diagram of our model (Eqs. (1), (2) and (3)). $\dot{\ell }$ represents the locomotion velocity (the tip expansion velocity), and *h*(*t*) represents a binary indicator of stimulation presence (h(t)=1) or absence (h(t)=0). A line with a circle represents an activation effect, a line with a T-shaped end represents an inhibition effect, and a double-headed arrow represents the diffusion effect

The variable ℓ represents the position of plasmodium tip $[\hbox {m}]$. The variables $p_{\textrm{f}}$ and $p_{\textrm{r}}$ represent the sol pressures [Pa] at the front and rear parts, respectively (Fig. 3(a)). Eqs. (2) and (3) are derived from a chemical reaction–diffusion system (see Sect. 3.2 for details). The terms $d(p_{\textrm{r}}-p_{\textrm{f}})$ and $r_{\textrm{v}}d(p_{\textrm{f}}-p_{\textrm{r}})$ are associated with the chemical diffusion between the front and rear parts, respectively. $r_{\textrm{v}}$ is their volume ratio, and *d* is related to the diffusion coefficient [1/s]. The terms $F_{\textrm{f}}(p_{\textrm{f}})$ and $F_{\textrm{r}}(p_{\textrm{r}})$ (Eqs. (4) and (5)) are associated with chemical reactions. $a_{\textrm{de}}$ and $a_{\textrm{de}}^{\textrm{s}}$ represent the chemical decomposition rate [1/s], and $g_{\textrm{f}}$, $g_{\textrm{f}}^{\textrm{s}}$ and $g_{\textrm{r}}$ are related to the generation of the chemicals [Pa/s]. Function *h*(*t*) equals 1 in the presence of stimulation and 0 in its absence. Here, the presence of stimulation denotes that plasmodium detects the repellent, whereas the absence denotes that it does not.

Plasmodium expands its tip through sol flow in the tube network. The sol flow is driven by the gradient of the sol pressure. Therefore, we assumed that the tip expansion velocity is proportional to the pressure gradient (Eq. (1)). In our model, we restricted the plasmodium’s locomotion to a straight line because extending the model to two dimensions would substantially increase its complexity. In this study, our primary focus is to understand the fundamental structure underlying habituation rather than the detailed spatial geometry of *Physarum*. Therefore, we limited the analysis to one-dimensional motion. In addition, we regarded plasmodium as consisting of two parts: the front part, which is the extendable tip for locomotion, and the rear part, which is the section opposite to the tip (Fig. 3(a)). Accordingly, we introduced the sol pressure variable at each part, $p_{\textrm{f}}$ and $p_{\textrm{r}}$. Thus, $p_{\textrm{r}}-p_{\textrm{f}}$ represents the pressure gradient (Eq. (1)). By identifying the sol pressure with the concentration of a relevant chemical species, we formulated the dynamics of the sol pressure using a simplified reaction–diffusion system (Eqs. (2) and (3)).

We developed a mathematical model describing plasmodium locomotion driven by a spatial difference in chemical concentration that regulates sol pressure. The input of stimulation directly influences the pressure at the front $p_{\textrm{f}}$, whereas the pressure at the rear $p_{\textrm{r}}$ indirectly affected through chemical diffusion (Fig. 3(b)). The tip expansion velocity (Eq. (1)) is determined by the difference between the pushing force from the rear $p_{\textrm{r}}$, which activates forward movement, and the pushing-back force from the front $p_{\textrm{f}}$, which inhibits it (Fig. 3(b)). In the following section, we describe the model formation in more detail.

### Detail of Formulation

First, we derived a locomotion model (Eq. (1)). We assumed that the tip expansion velocity was proportional to the volumetric flow rate of the sol toward its tip because plasmodium expanded its tip due to the sol flow in the tube network. Thus, we formulated the tip expansion velocity (hereafter called the locomotion velocity) as follows:

$$\begin{aligned} \frac{d\ell }{dt}(t)=k_{\ell }Q(t), \end{aligned}$$

where *Q*(*t*) denotes the volumetric flow rate of the sol [$\hbox {m}^{3}$/s], and $k_{\ell }$ denotes positive constant [1/$\hbox {m}^2$]. The sol flow inside the tube can be approximated using a Hagen-Poiseuille flow. The volumetric flow rate is expressed as follows:

$$\begin{aligned} Q = \frac{\pi R^4}{8\mu L}(p_{1}-p_{2}), \end{aligned}$$

where *Q* is the volumetric flow rate, *R* and *L* are the tube radius and length [m], μ is the viscosity coefficient [Pa · s], and $p_1$ and $p_2$ denote the pressures at the tube edges [Pa]. In this study, we considered plasmodium to be composed of two parts: front and rear (Fig. 3(a)). We assumed that *n* tubes cross between the front and rear parts and that they are identical. Therefore, the volumetric flow rate of the sol toward the front part is as follows:

$$\begin{aligned} Q(t) = n \frac{\pi R^4}{8\mu L}(p_{\textrm{r}}(t)-p_{\textrm{f}}(t)). \end{aligned}$$

Consequently, the locomotion velocity of plasmodium is represented as

$$\begin{aligned} \frac{d\ell }{dt}(t) =\gamma (p_{\textrm{r}}(t)-p_{\textrm{f}}(t)), \end{aligned}$$

where γ denotes positive constant [m/Pa · s] satisfying

$$\begin{aligned} \gamma = k_{\ell }n \frac{\pi R^4}{8\mu L}. \end{aligned}$$

Next, we formulated a reaction–diffusion model for chemically involved gel contraction with simplified diffusion terms. We considered the chemical reactions, generation and decomposition, and the chemical diffusion between the front and rear parts. Then, the reaction–diffusion model was formulated as follows:

6 $$\begin{aligned} \frac{dc_{\textrm{f}}}{dt}(t)&= d(c_{\textrm{r}}-c_{\textrm{f}}) + F_{\textrm{f}}(c_{\textrm{f}}) , \end{aligned}$$

7 $$\begin{aligned} \frac{dc_{\textrm{r}}}{dt}(t)&= r_{\textrm{v}}d(c_{\textrm{f}}-c_{\textrm{r}}) + F_{\textrm{r}}(c_{\textrm{r}}), \end{aligned}$$

where

$$\begin{aligned} F_{\textrm{f}}(c_{\textrm{f}})&= (1-h(t))(-a_{\textrm{de}}c_{\textrm{f}}+g_{\textrm{f}}) +h(t)(-a_{\textrm{de}}^{\textrm{s}}c_{\textrm{f}}+g_{\textrm{f}}^{\textrm{s}}), \\ F_{\textrm{r}}(c_{\textrm{r}})&= -a_{\textrm{de}}c_{\textrm{r}}+g_{\textrm{r}}. \end{aligned}$$

The variables $c_{\textrm{f}}$ and $c_{\textrm{r}}$ represent the concentrations of a chemical involved in gel contraction in the front and rear parts [mol/$\hbox {m}^3$], respectively. The terms $d(c_{\textrm{r}}-c_{\textrm{f}})$ and $r_{\textrm{v}}d(c_{\textrm{f}}-c_{\textrm{r}})$ represent the chemical diffusion between the front and rear parts, respectively, where $r_{\textrm{v}}$ is their volume ratio, and *d* is related to the diffusion coefficient [1/s]. Note that we assumed diffusion between solutions with different volumes, and therefore diffusion terms describe above (Appendix A). The terms $F_{\textrm{f}}(c_{\textrm{f}})$ and $F_{\textrm{r}}(c_{\textrm{r}})$ represent chemical reactions. $-a_{\textrm{de}}c_{\textrm{f}}$, $-a_{\textrm{de}}^{\textrm{s}}c_{\textrm{f}}$ and $-a_{\textrm{de}}c_{\textrm{r}}$ represent the decomposition of chemicals, where $a_{\textrm{de}}$ and $a_{\textrm{de}}^{\textrm{s}}$ represent the chemical decomposition rate [1/s]. $g_{\textrm{f}}$, $g_{\textrm{f}}^{\textrm{s}}$ and $g_{\textrm{r}}$ represent the generation of the chemicals [mol/$\hbox {m}^3$ · s]. Function *h*(*t*) equals 1 in the presence of stimulation and 0 in its absence. The presence of stimulation denotes that plasmodium detects the repellent, whereas the absence denotes that it does not. Here, the chemical reaction at the front switches depending on its presence or absence because plasmodium detects the repellent at the front.

Finally, we derived the temporal dynamics of the sol pressure. Sol pressure was generated by gel contraction. In this study, we assumed a linear relationship between sol pressure and the concentration of a chemical involved in gel contraction, although a more complex relationship may exist. We will discuss biological background of this assumption in more detail later in Discussion Section (Sect. 6.4). From this assumption, the following equations were obtained:

8 $$\begin{aligned} p_{\textrm{f}}(t) = kc_{\textrm{f}}(t), p_{\textrm{r}}(t) = kc_{\textrm{r}}(t), \end{aligned}$$

where *k* denotes a constant value. From Eqs. (6), (7), and (8), the sol-pressure dynamics were derived as follows:

$$\begin{aligned} \frac{dp_{\textrm{f}}}{dt}(t)&= d(p_{\textrm{r}}-p_{\textrm{f}}) + F_{\textrm{f}}(p_{\textrm{f}}),\\ \frac{dp_{\textrm{r}}}{dt}(t)&= r_{\textrm{v}}d(p_{\textrm{f}}-p_{\textrm{r}}) + F_{\textrm{r}}(p_{\textrm{r}}), \end{aligned}$$

where

$$\begin{aligned} F_{\textrm{f}}(p_{\textrm{f}})&= (1-h(t))(-a_{\textrm{de}}p_{\textrm{f}}+g_{\textrm{f}}) +h(t)(-a_{\textrm{de}}^{\textrm{s}}p_{\textrm{f}}+g_{\textrm{f}}^{\textrm{s}}), \\ F_{\textrm{r}}(p_{\textrm{r}})&= -a_{\textrm{de}}p_{\textrm{r}}+g_{\textrm{r}}. \end{aligned}$$

Here, $kg_{\textrm{f}}$, $kg_{\textrm{f}}^{\textrm{s}}$ and $kg_{\textrm{r}}$ replace $g_{\textrm{f}}$, $g_{\textrm{f}}^{\textrm{s}}$ and $g_{\textrm{r}}$ [Pa/s], respectively. In conclusion, the model presented in Sect. 3.1 is obtained.

**Fig. 4:**
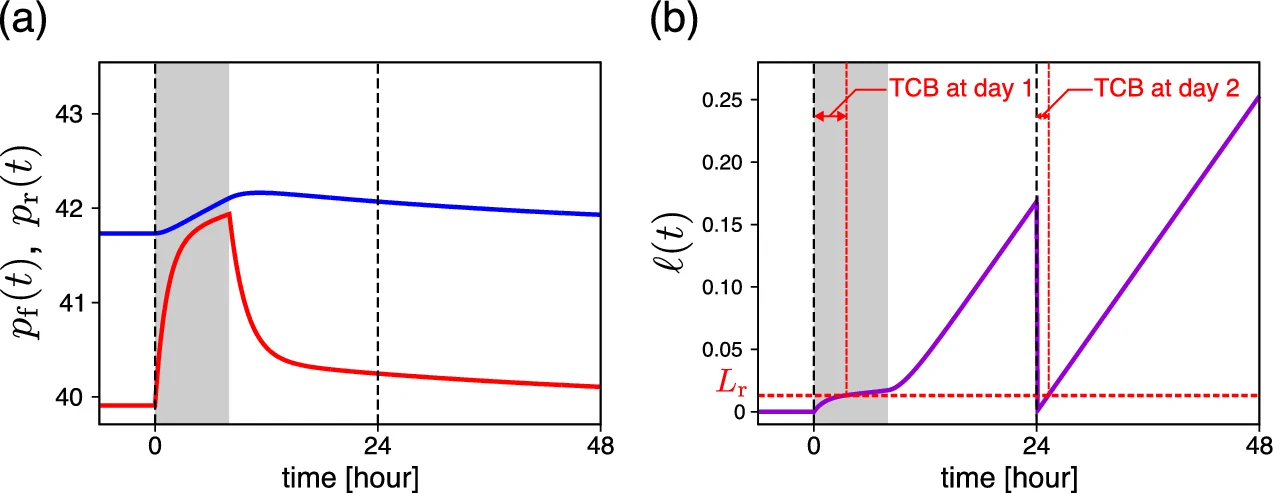
Time course of our model (Eqs. (1), (2), and (3)) where the bridge condition at day 1 is the b-Q, and that at day 2 is the b-N. Gray background represents the term in the presence of stimulation, h(t)=1, and white background represents the term in the absence of stimulation, h(t)=0. (a) Time course of sol pressure. The red line represents $p_{\textrm{f}}$, and the blue line represents $p_{\textrm{r}}$. (b) Time course of the tip position of plasmodium. The purple line represents ℓ, The value of ℓ resets to 0 at the start of the day. $L_{\textrm{r}}$ represents the bridge length. TCB means the time to cross the bridge

### Numerical Simulations

We explain the simulation settings using a simple case study (Fig. 4). The purpose of our simulation was to compute the time required to cross the bridge daily for 9 days, as in Boisseau et al. (2016). In the following simulations, time is measured in hours. First, given the initial value of $(p_{\textrm{f}}(t), p_{\textrm{r}}(t))$ for each day, we compute the crossing time using Eqs. (1), (2), and (3) (Fig. 4). We applied stimulation in the following pattern, depending on the bridge type. If the bridge type is the b-Q on day *n* $(t_{n-1}\le t < t_{n})$,

$$\begin{aligned} h(t)&= {\left\{ \begin{array}{ll} 1, & (t_{n-1}\le t< t_{n-1}+T_{\textrm{s}}),\\ 0, & (t_{n-1}+T_{\textrm{s}}\le t < t_{n}), \end{array}\right. } \end{aligned}$$

where $t_n=Tn$, $0<T_{\textrm{s}}<T$. $T_{\textrm{s}}$ denotes the duration of stimulation, that is, the period during which the repellent is detected. *T* denotes the duration of one period, which was set to T=24 here. If the bridge is the b-N on day *n*,

$$\begin{aligned} h(t)&=0, \ (t_{n-1}\le t < t_{n}). \end{aligned}$$

The initial value of $(p_{\textrm{f}}(t), p_{\textrm{r}}(t))$ on Day 1 is $(P_{\textrm{f}}^{\textrm{u}}, P_{\textrm{r}}^{\textrm{u}})$, which is the equilibrium state in the absence of stimulation. After Day 2, the final states of $(p_{\textrm{f}}(t), p_{\textrm{r}}(t))$ on the previous day were used as the initial conditions (Fig. 4(a)). Second, we computed the time to cross the bridge as the time required for ℓ(t), the position of plasmodium tips, to reach $L_{\textrm{r}}$, the length of the bridge [m], on each day (Fig. 4(b)).

**Fig. 5:**
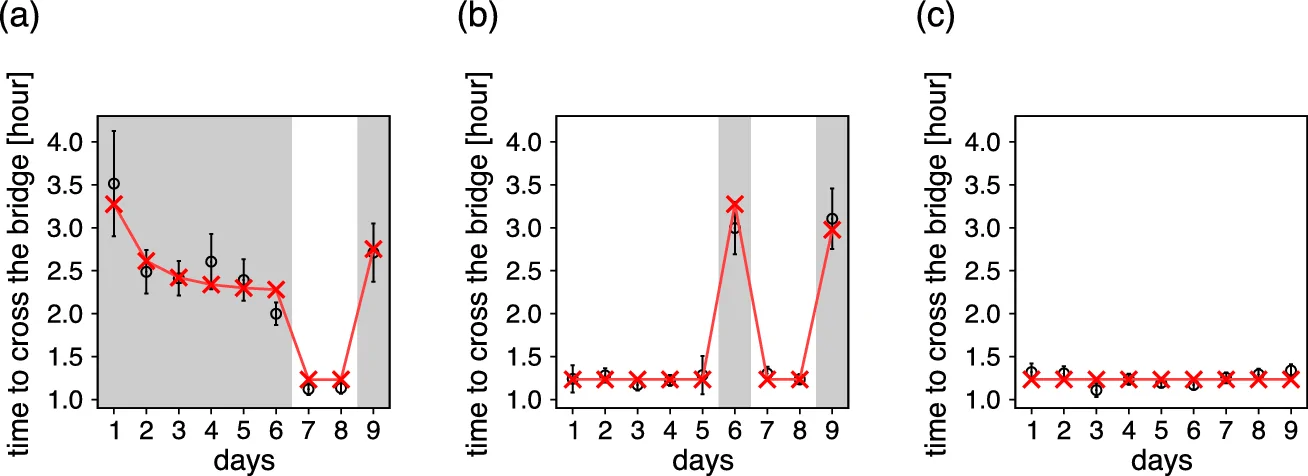
Our simulation results of time to cross the bridge using the best-fit parameter set. The red cross mark represents our simulation results. The circle represents the mean time to cross the bridge in the experiment (Boisseau et al. 2016), and the error bar represents the 95 percent confidence interval. Gray and white backgrounds indicate the b-Q and b-N conditions, respectively. The error measured by modified Root Mean Squared Error (Eq. (9)) is $0.9076 \, \text {[h]}$. (Parameters: $0\le t \le 216 \, \text {[h]}$, $L_{\textrm{r}}=1.300\times 10^{-2} \, \text {[m]}$, $T_{\textrm{s}}=8.0 \, \text {[h]}$, $T=24.0 \, \text {[h]}$, $g_{\textrm{r}}=1.000$[Pa/h], $g_{\textrm{f}}=1.001\times 10^{-3}$[Pa/h], $g_{\textrm{f}}^{\textrm{s}}=1.197\times 10$[Pa/h], $a_{\textrm{de}}=2.244\times 10^{-2}$ [1/h], $a_{\textrm{de}}^{\textrm{s}}=2.866\times 10^{-1}$[1/h], $d=4.909\times 10^{-1}$[1/h], $\gamma =5.782\times 10^{-3}$[m/Pa · h], $r_{\textrm{v}}=7.088\times 10^{-2})$

We conducted the parameter estimation by fixing $g_{\textrm{r}}=1$ via non-dimensionalization (see Online Resource 1). Our simulation results using the best parameter set reproduced the three features (i) – (iii) observed experimentally (Fig. 5), with an error of 0.9076 [h]. Here, we used the modified Root Mean Squared Error (*RMSE*), defined as follows:

9 $$\begin{aligned} RMSE = \sqrt{ \frac{1}{3D} \sum _{i\in \{\textrm{a}, \textrm{b}, \textrm{c}\}}\sum _{j=1}^{D} \frac{1}{N_{i}} \sum _{k=1}^{N_{i}} (T_{j}^{i}(\boldsymbol{\theta })-T_{jk}^{i})^2 } \end{aligned}$$

where the index *i* denotes the dataset group (a, b and c correspond to the dataset Fig. 2(a), (b) and (c), respectively), the index *j* represents day, and the index *k* denotes the each individual. $N_i$ represents the number of samples in group *i*, and *D* represents the total days of experiment. $T_{j}^{i}(\boldsymbol{\theta })$ and $T_{jk}^{i}$ represent the bridge-crossing time obtained from the model simulation with parameters $\boldsymbol{\theta }= (g_{\textrm{f}}, g_{\textrm{f}}^{\textrm{s}}, a_{\textrm{de}}, a_{\textrm{de}}^{\textrm{s}}, d, \gamma , r_{\textrm{v}})^t$ and the experimental measurements, respectively.

**Fig. 6:**
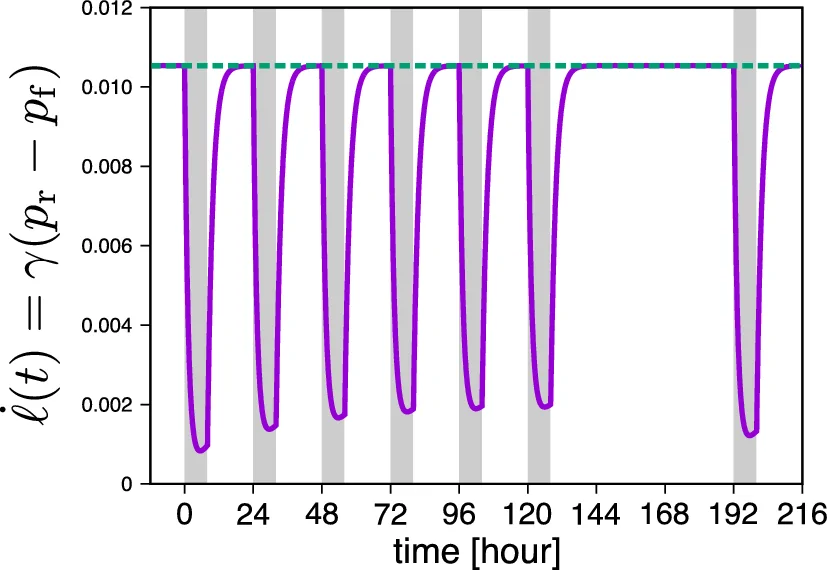
The temporal dynamics of the tip expansion velocity corresponding to Fig. 5(a). Gray background represents the term under stimulation, h(t)=1, white background represents the term in the absence of stimulation, h(t)=0, and green broken line represents that the velocity is equal to $\gamma (P_{\textrm{r}}^{\textrm{u}}-P_{\textrm{f}}^{\textrm{u}})$

We focused on the temporal dynamics of the tip expansion velocity, corresponding to Fig. 5(a) (Fig. 6). Mitigation of deceleration due to repeated stimuli, followed by recovery of deceleration after a temporary interruption in stimulation, was observed. In addition, the velocity immediately returned to $\gamma (P_{\textrm{r}}^{\textrm{u}}-P_{\textrm{f}}^{\textrm{u}})$ when the stimulation was withheld (Fig. 6). The mitigation of deceleration under stimulation indicated a decline in the time to cross the b-Q, and its recovery indicated an increase in the time to cross the b-Q (Fig. 5(a)). In addition, the immediate return of velocity to $\gamma (P_{\textrm{r}}^{\textrm{u}}-P_{\textrm{f}}^{\textrm{u}})$ corresponds to the consistency of the time to cross the b-N (Fig. 5). In other words, these temporal behaviors enable the reproduction of three features (i) ∼ (iii). Therefore, in the subsequent section, we discuss the relationship between the sol pressures $(p_{\textrm{f}}, p_{\textrm{r}})$ and the velocity dynamics ($N_{\textrm{a}}=68$, $N_{\textrm{b}}=82$, $N_{\textrm{c}}=100$, D=9).

## Mathematical Analysis

We analyzed the behavior of the locomotion velocity from the behavior of the sol pressure on the $(p_{\textrm{f}}, p_{\textrm{r}})$ plane. At this point, the dynamics of the sol pressure in the presence and absence of stimulation on the $(p_{\textrm{f}}, p_{\textrm{r}})$ plane and the relationship between the pressure and locomotion output are required. In Sect. 4.1, we analyze the dynamics of the pressure on the $(p_{\textrm{f}}, p_{\textrm{r}})$ plane and the relationship between the pressure and locomotion output. In Sect. 4.2 and 4.3, we discuss the behavior of the locomotion velocity based on the analysis in Sect. 4.1.

### Mathematical Preparation

First, we analyzed the dynamics of the sol pressure. In the presence of a stimulation (h(t)=1), Eqs. (2) and (3) can be transformed as follows.

10 $$\begin{aligned} \frac{d}{dt} \begin{pmatrix} p_{\textrm{f}}\\ p_{\textrm{r}}\end{pmatrix}= \begin{pmatrix} -(a_{\textrm{de}}^{\textrm{s}}+d) & d \\ r_{\textrm{v}}d & -(a_{\textrm{de}}+r_{\textrm{v}}d) \end{pmatrix} \begin{pmatrix} p_{\textrm{f}}- P_{\textrm{f}}^{\textrm{s}}\\ p_{\textrm{r}}- P_{\textrm{r}}^{\textrm{s}}\end{pmatrix}, \end{aligned}$$

where $(P_{\textrm{f}}^{\textrm{s}}, P_{\textrm{r}}^{\textrm{s}})$ denotes the equilibrium point.

$$\begin{aligned} P_{\textrm{f}}^{\textrm{s}}= \frac{(a_{\textrm{de}}+r_{\textrm{v}}d)g_{\textrm{f}}^{\textrm{s}}+ dg_{\textrm{r}}}{a_{\textrm{de}}^{\textrm{s}}(a_{\textrm{de}}+r_{\textrm{v}}d)+a_{\textrm{de}}d}, P_{\textrm{r}}^{\textrm{s}}= \frac{r_{\textrm{v}}dg_{\textrm{f}}^{\textrm{s}}+ (a_{\textrm{de}}^{\textrm{s}}+d)g_{\textrm{r}}}{a_{\textrm{de}}^{\textrm{s}}r_{\textrm{v}}d+a_{\textrm{de}}(a_{\textrm{de}}^{\textrm{s}}+d)}. \end{aligned}$$

The eigenvalues and eigenvectors in Eq. (10) are

11 $$\begin{aligned} \lambda _{1}&= \frac{-B-\sqrt{D}}{2}, \ \boldsymbol{v}_{1} = \begin{pmatrix} \lambda _{1} +a_{\textrm{de}}+r_{\textrm{v}}d \\ r_{\textrm{v}}d \end{pmatrix},\end{aligned}$$

12 $$\begin{aligned} \lambda _{2}&= \frac{-B+\sqrt{D}}{2}, \ \boldsymbol{v}_{2} = \begin{pmatrix} d \\ \lambda _{2} +a_{\textrm{de}}^{\textrm{s}}+d \end{pmatrix} , \end{aligned}$$

where $\lambda _{i}$ represents the eigenvalue, $\boldsymbol{v}_{i}$ represents the eigenvector corresponding to eigenvalue $\lambda _{i}$, and

$$\begin{aligned} B&= a_{\textrm{de}}^{\textrm{s}}+ a_{\textrm{de}}+ d +r_{\textrm{v}}d, \\ D&= (a_{\textrm{de}}^{\textrm{s}}- a_{\textrm{de}}+ d -r_{\textrm{v}}d)^2 +4r_{\textrm{v}}d^2. \end{aligned}$$

Thus, the solution to Eq. (10) is given as follows:

13 $$\begin{aligned} \begin{pmatrix} p_{\textrm{f}}(t)\\ p_{\textrm{r}}(t) \end{pmatrix}= \begin{pmatrix} P_{\textrm{f}}^{\textrm{s}}\\ P_{\textrm{r}}^{\textrm{s}}\end{pmatrix}+P \begin{pmatrix} e^{\lambda _1(t-t_0)} & \quad 0 \\ 0 & \quad e^{\lambda _2(t-t_0)} \end{pmatrix} P^{-1} \begin{pmatrix} p_{\textrm{f}}(t_0)-P_{\textrm{f}}^{\textrm{s}}\\ p_{\textrm{r}}(t_0)-P_{\textrm{r}}^{\textrm{s}}\end{pmatrix} , \end{aligned}$$

where $t_0$ denotes the initial time,

$$\begin{aligned} P&= \begin{pmatrix} \lambda _{1} +a_{\textrm{de}}+r_{\textrm{v}}d & d\\ r_{\textrm{v}}d & \lambda _{2} +a_{\textrm{de}}^{\textrm{s}}+d \end{pmatrix}, \\ P^{-1}&=-\frac{2}{D+(a_{\textrm{de}}^{\textrm{s}}-a_{\textrm{de}}+d-r_{\textrm{v}}d)\sqrt{D}} \begin{pmatrix} \lambda _{2} +a_{\textrm{de}}^{\textrm{s}}+d & -d\\ -r_{\textrm{v}}d & \lambda _{1} +a_{\textrm{de}}+r_{\textrm{v}}d \end{pmatrix}. \end{aligned}$$

**Fig. 7:**
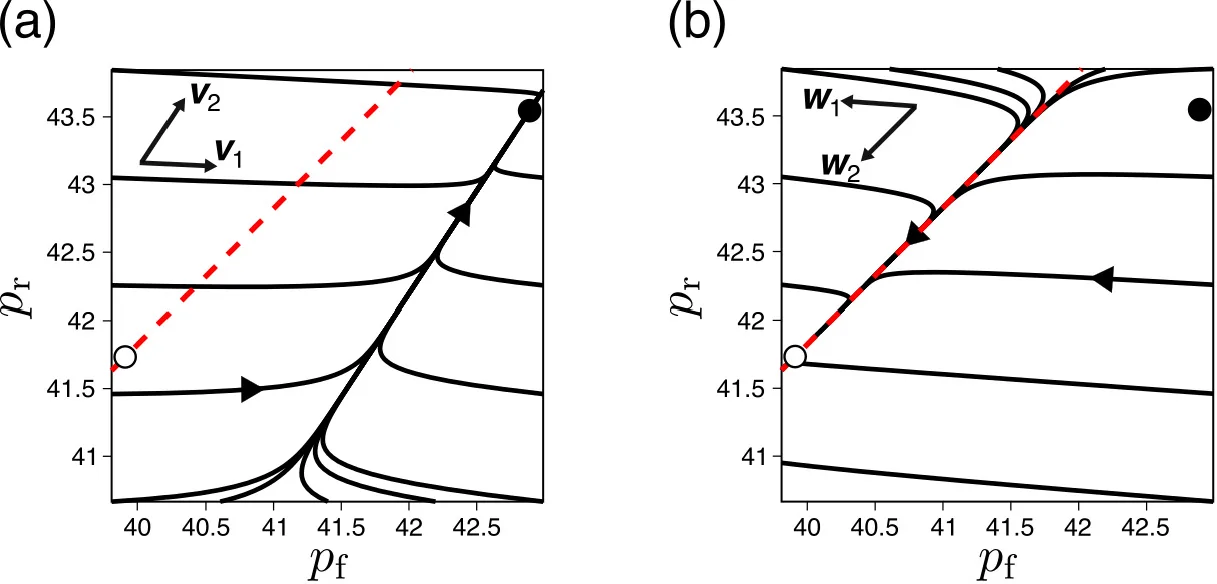
The solution trajectory of Eqs. (10) and (14). The red broken line represents the straight line $p_{\textrm{r}}=p_{\textrm{f}}+P_{\textrm{r}}^{\textrm{u}}-P_{\textrm{f}}^{\textrm{u}}$. The white circle represents the equilibrium point in the absence of stimulation, $(P_{\textrm{f}}^{\textrm{u}}, P_{\textrm{r}}^{\textrm{u}})$, and the black circle represents the equilibrium point under stimulation, $(P_{\textrm{f}}^{\textrm{s}}, P_{\textrm{r}}^{\textrm{s}})$. (a) The solution trajectory of Eq. (10). The arrows at top-left represent the directions of eigenvector $\boldsymbol{v}_{1}$ and $\boldsymbol{v}_{2}$ (Eqs. (11) and (12)). (b) The solution trajectory of Eq. (14). The arrows at top-left represent the directions of eigenvector $\boldsymbol{w}_{1}$ and $\boldsymbol{w}_{2}$ (Eqs. (15) and (16)). The parameters are the same at Fig. 5.

The dynamics of Eq. (13) on the phase plane are depicted in Fig. 7(a). In the absence of a stimulation (h(t)=0), Eq. (2) and (3) can be modified as follows:

14 $$\begin{aligned} \frac{d}{dt} \begin{pmatrix} p_{\textrm{f}}\\ p_{\textrm{r}}\end{pmatrix}= \begin{pmatrix} -(a_{\textrm{de}}+d) & d \\ r_{\textrm{v}}d & -(a_{\textrm{de}}+r_{\textrm{v}}d) \end{pmatrix} \begin{pmatrix} p_{\textrm{f}}- P_{\textrm{f}}^{\textrm{u}}\\ p_{\textrm{r}}- P_{\textrm{r}}^{\textrm{u}}\end{pmatrix}, \end{aligned}$$

where $(P_{\textrm{f}}^{\textrm{u}}, P_{\textrm{r}}^{\textrm{u}})$ denotes the equilibrium point.

$$\begin{aligned} P_{\textrm{f}}^{\textrm{u}}= \frac{(a_{\textrm{de}}+r_{\textrm{v}}d)g_{\textrm{f}}+ dg_{\textrm{r}}}{a_{\textrm{de}}(a_{\textrm{de}}+r_{\textrm{v}}d+d)}, P_{\textrm{r}}^{\textrm{u}}= \frac{r_{\textrm{v}}dg_{\textrm{f}}+ (a_{\textrm{de}}+d)g_{\textrm{r}}}{a_{\textrm{de}}(r_{\textrm{v}}d+a_{\textrm{de}}+d)}. \end{aligned}$$

The eigenvalues and eigenvectors in Eq. (14) are

15 $$\begin{aligned} \mu _1&= -(a_{\textrm{de}}+d+r_{\textrm{v}}d), \ \boldsymbol{w}_1 = \begin{pmatrix} 1 \\ -r_{\textrm{v}}\end{pmatrix}, \end{aligned}$$

16 $$\begin{aligned} \mu _2&= -a_{\textrm{de}}, \ \boldsymbol{w}_2 = \begin{pmatrix} 1 \\ 1 \end{pmatrix}, \end{aligned}$$

where $\mu _{i}$ represents the eigenvalue, and $\boldsymbol{w}_{i}$ represents the eigenvector corresponding to the eigenvalue $\mu _{i}$. Thus, the solution to Eq. (14) is given as follows:

17 $$\begin{aligned} \begin{pmatrix} p_{\textrm{f}}(t)\\ p_{\textrm{r}}(t) \end{pmatrix}= \begin{pmatrix} P_{\textrm{f}}^{\textrm{s}}\\ P_{\textrm{r}}^{\textrm{s}}\end{pmatrix}+Q \begin{pmatrix} e^{\mu _1(t-t_0)} & 0 \\ 0 & e^{\mu _2(t-t_0)} \end{pmatrix}Q^{-1} \begin{pmatrix} p_{\textrm{f}}(t_0)-P_{\textrm{f}}^{\textrm{u}}\\ p_{\textrm{r}}(t_0)-P_{\textrm{r}}^{\textrm{u}}\end{pmatrix} , \end{aligned}$$

where $t_0$ denotes the initial time,

$$\begin{aligned} Q&= \begin{pmatrix} 1 & 1 \\ -r_{\textrm{v}}& 1 \end{pmatrix}, \\ Q^{-1}&=\frac{1}{1+r_{\textrm{v}}} \begin{pmatrix} 1 & -1 \\ r_{\textrm{v}}& 1 \end{pmatrix}. \end{aligned}$$

The dynamics of Eq. (17) on the phase plane are depicted in Fig. 7(b).

**Fig. 8:**
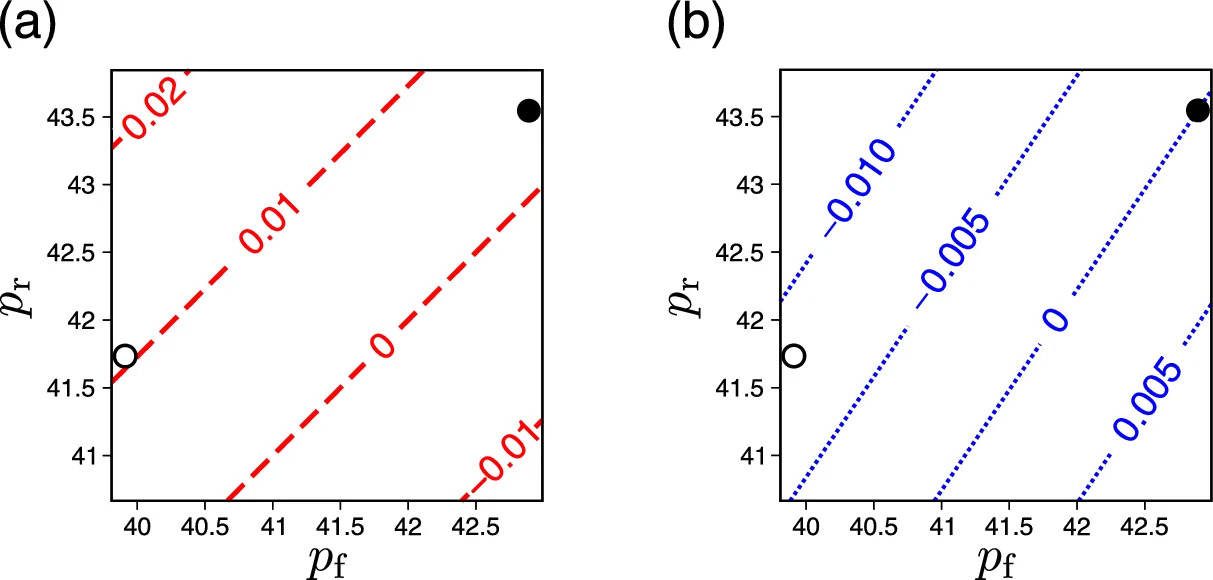
The relationship between the sol pressure $(p_{\textrm{f}}, p_{\textrm{r}})$ and behavioral output on the $(p_{\textrm{f}}, p_{\textrm{r}})$ plane. The white circle represents $(P_{\textrm{f}}^{\textrm{u}}, P_{\textrm{r}}^{\textrm{u}})$, and the black circle represents $(P_{\textrm{f}}^{\textrm{s}}, P_{\textrm{r}}^{\textrm{s}})$. (a) The plot of $V(p_{\textrm{f}}, p_{\textrm{r}})$ on the $(p_{\textrm{f}}, p_{\textrm{r}})$ plane. Red broken lines represent the iso-velocity lines. The value on each line represents the velocity corresponding to the sol pressure $(p_{\textrm{f}}, p_{\textrm{r}})$ on the line. (b) The plot of $A_\textrm{s}(p_{\textrm{f}}, p_{\textrm{r}})$ on the $(p_{\textrm{f}}, p_{\textrm{r}})$ plane. Blue dotted lines represent the iso-acceleration lines. The value on each line represents the acceleration under stimulation corresponding to the sol pressure $(p_{\textrm{f}}, p_{\textrm{r}})$ on the line. The parameters are same at Fig. 5

Second, we analyzed the relationship between the sol pressure $(p_{\textrm{f}}, p_{\textrm{r}})$ and behavioral output on the $(p_{\textrm{f}}, p_{\textrm{r}})$ plane. From Eq. (1), we define the function $V(p_{\textrm{f}}, p_{\textrm{r}})$ that returns the locomotion velocity corresponding to the sol pressure $(p_{\textrm{f}}, p_{\textrm{r}})$.

18 $$\begin{aligned} V(p_{\textrm{f}}, p_{\textrm{r}}) = \gamma (p_{\textrm{r}}-p_{\textrm{f}}). \end{aligned}$$

From Eq. (18), $V(p_{\textrm{f}}, p_{\textrm{r}})$ has a constant value on the line

19 $$\begin{aligned} p_{\textrm{r}}=p_{\textrm{f}}+(\textrm{const}). \end{aligned}$$

For example, $V(p_{\textrm{f}}, p_{\textrm{r}})=1$ for all $(p_{\textrm{f}}, p_{\textrm{r}})$ satisfying $p_{\textrm{r}}=p_{\textrm{f}}+1/\gamma$. The lines in Eq. (19) is referred to as the iso-velocity line. The iso-velocity lines are shown in Fig. 8(a). Moreover, by differentiating both sides of Eq. (1), and substituting Eq. (10), we obtain the acceleration under stimulation as follows:

$$\begin{aligned} \frac{d^2 \ell }{dt^2}(t)=-\gamma (a_{\textrm{de}}+d +r_{\textrm{v}}d)\left( p_{\textrm{r}}(t) - \frac{a_{\textrm{de}}^{\textrm{s}}+d +r_{\textrm{v}}d}{a_{\textrm{de}}+d +r_{\textrm{v}}d}p_{\textrm{f}}(t)\right) -\gamma (g_{\textrm{f}}^{\textrm{s}}-g_{\textrm{r}}). \end{aligned}$$

We define the function $A_\textrm{s}(p_{\textrm{f}}, p_{\textrm{r}})$, which returns the acceleration under stimulation for sol pressure $(p_{\textrm{f}}, p_{\textrm{r}})$.

20 $$\begin{aligned} A_\textrm{s}(p_{\textrm{f}}, p_{\textrm{r}})=-\gamma (a_{\textrm{de}}+d +r_{\textrm{v}}d)\left( p_{\textrm{r}}- \frac{a_{\textrm{de}}^{\textrm{s}}+d +r_{\textrm{v}}d}{a_{\textrm{de}}+d +r_{\textrm{v}}d}p_{\textrm{f}}\right) -\gamma (g_{\textrm{f}}^{\textrm{s}}-g_{\textrm{r}}). \end{aligned}$$

From Eq. (20), $A_\textrm{s}(p_{\textrm{f}}, p_{\textrm{r}})$ has a constant value on the line

21 $$\begin{aligned} p_{\textrm{r}}= \frac{a_{\textrm{de}}^{\textrm{s}}+d +r_{\textrm{v}}d}{a_{\textrm{de}}+d +r_{\textrm{v}}d}p_{\textrm{f}}+(\textrm{const}). \end{aligned}$$

**Fig. 9:**
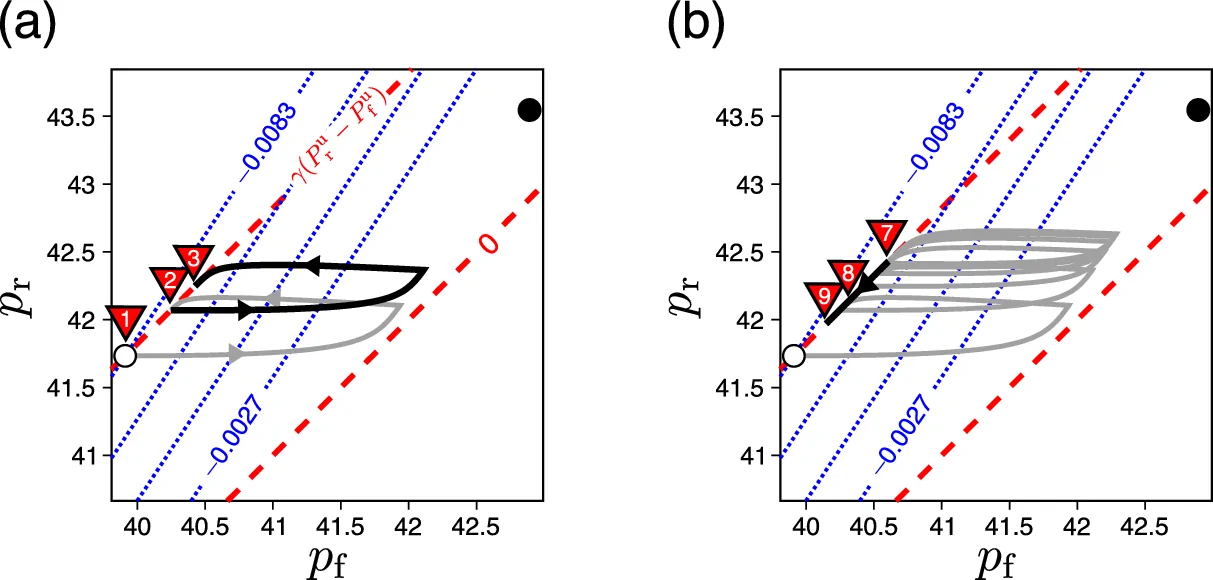
The sol pressure dynamics on the $(p_{\textrm{f}}, p_{\textrm{r}})$ plane corresponding to Fig. 5(a). The white circle represents $(P_{\textrm{f}}^{\textrm{u}}, P_{\textrm{r}}^{\textrm{u}})$, the black circle represents $(P_{\textrm{f}}^{\textrm{s}}, P_{\textrm{r}}^{\textrm{s}})$, red broken lines represent the iso-velocity lines, blue dotted lines represent the iso-acceleration lines, and the red triangle with integer *n* points to the initial state at day *n*. (a) The trajectory of day 1∼2: the gray solid line represents the trajectory of day 1 (b-Q), and the black solid line represents the trajectory of day 2 (b-Q). (b) The trajectory of day 1∼8: the gray solid line represents the trajectory of day 1∼6 (b-Q), and the black solid line represents the trajectory of day 7∼8 (b-N)

The lines in Eq. (21) is referred to as the iso-acceleration line. The iso-acceleration lines are shown in Fig. 8(b). Because $a_{\textrm{de}}^{\textrm{s}}>a_{\textrm{de}}>0$ holds for our simulation parameters, the slope of the iso-acceleration line is greater than 1 (Eq. (21)). Hence, the iso-acceleration and iso-velocity lines intersect, as shown in Fig. 9.

### Mitigation and Recovery of Deceleration

In this section, we discuss the mitigation and recovery of the deceleration under stimulation. The shift in the initial pressure state each day was crucial for the mitigation and recovery of deceleration under stimulation. By repeatedly crossing the b-Q, the initial state of each day shifts to the upper right (Fig. 9(a)). Focusing on the iso-acceleration lines, the initial state transitions in the direction in which the acceleration increases (Fig. 9(a)). It follows that the deceleration is mitigated (Fig. 6). On the other hand, the initial state shifted to the lower left each day when crossing the b-N (Fig. 9(b)). In other words, the initial state transitions in the opposite direction when repeatedly crossing the b-Q. Thus, deceleration under stimulation recovered when crossing the b-N. In summary, the mitigation of deceleration when repeatedly crossing the b-Q corresponded to an upper right shift of the initial state (Fig. 9(a)), and its recovery when crossing the b-N corresponded to a lower left shift (Fig. 9(b)).

Next, we discuss why the initial state shift occurs when the crossing the b-Q is repeated. To focus on the displacement in the direction along the iso-velocity line, namely, $(1,1)^t$, we introduce the following variable:

$$\begin{aligned} m(t)=p_{\textrm{r}}(t)+p_{\textrm{f}}(t). \end{aligned}$$

The variable *m*(*t*) represents the location in the direction of $(1,1)^t$ on the $(p_{\textrm{f}}, p_{\textrm{r}})$ plane. From the temporal change in *m*(*t*), we can observe the temporal change in the coordinates on the axis parallel to the iso-velocity lines.

**Fig. 10:**
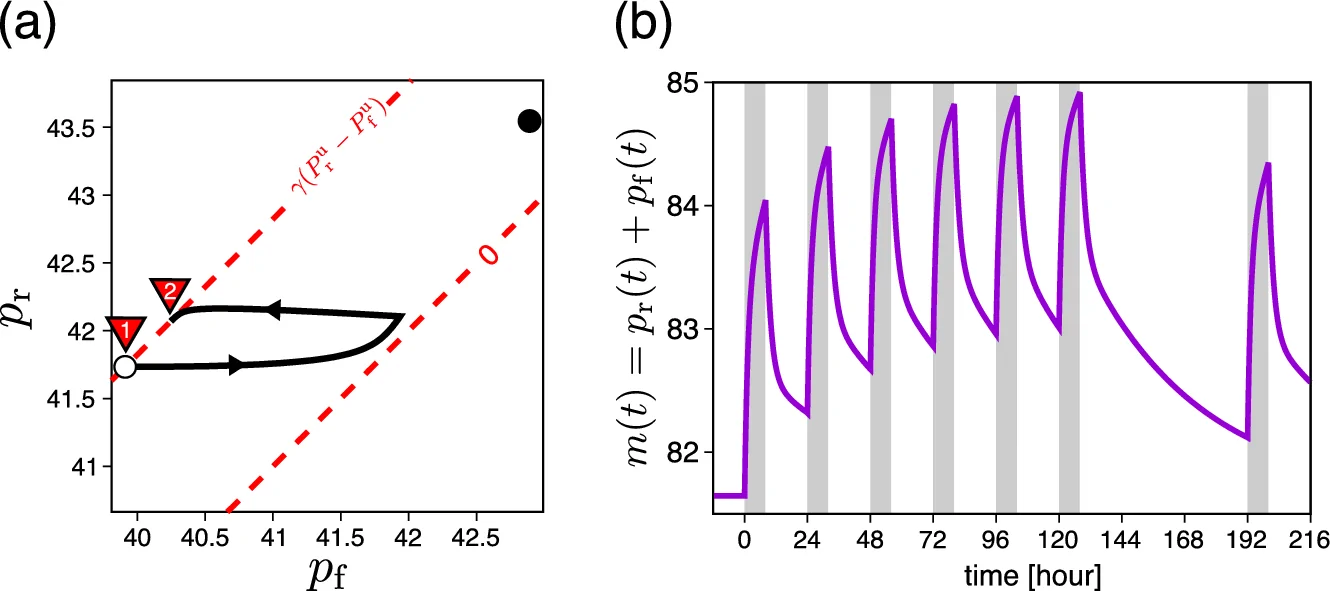
(a) The trajectory of day 1 on the $(p_{\textrm{f}}, p_{\textrm{r}})$ plane. The white circle represents $(P_{\textrm{f}}^{\textrm{u}}, P_{\textrm{r}}^{\textrm{u}})$, and the black circle represents $(P_{\textrm{f}}^{\textrm{s}}, P_{\textrm{r}}^{\textrm{s}})$. The red triangle with integer *n* points to the initial value at day *n*. (b) The temporal dynamics of *m*(*t*). Gray background represents the period under stimulation, h(t)=1, and white background represents the period without stimulation, h(t)=0

We focused on the pressure dynamics on day 1 when crossing the b-Q. The initial period of the day was during stimulation, followed by a period without stimulation. It follows that the pressure transitions toward the black point, $(P_{\textrm{f}}^{\textrm{s}}, P_{\textrm{r}}^{\textrm{s}})$, firstly, and then toward the white point, $(P_{\textrm{f}}^{\textrm{u}}, P_{\textrm{r}}^{\textrm{u}})$ (Fig. 10(a)). Considering the behavior of *m*(*t*), the increase in *m*(*t*) under stimulation exceeds its decrease in the absence of stimulation when 0≤t<24 (Fig. 10(b)). Consequently, the initial state on Day 2 was positioned to the upper right of that on Day 1 (Fig. 10(a)). Next, we focus on the case where the crossing the b-Q is repeated. The increase in *m*(*t*) under stimulation gradually balanced its decrease in the absence of stimulation by repeatedly crossing the b-Q (Fig. 10(b)). Consequently, the displacement of the initial state to the upper right decreased and the deceleration converged (Fig. 6).

In summary, a shift in the initial state of each day is important for mitigation and recovery from deceleration. Additionally, we found that its mitigation caused a difference between the displacement to the axis parallel to the iso-velocity lines under stimulation and in its absence.

### Velocity consistency on the b-N

Here, we discuss the reason for the constant locomotion velocity of the b-N. The initial locomotion velocity on each day was almost the same as $\gamma (P_{\textrm{r}}^{\textrm{u}}-P_{\textrm{f}}^{\textrm{u}})$ regardless of whether the bridge condition on the previous day was the b-Q or b-N (Fig. 6). In short, the initial states were sufficiently close to the straight line $p_{\textrm{r}}=p_{\textrm{f}}+P_{\textrm{r}}^{\textrm{u}}-P_{\textrm{f}}^{\textrm{u}}$, where the velocity was $\gamma (P_{\textrm{r}}^{\textrm{u}}-P_{\textrm{f}}^{\textrm{u}})$ (Fig. 9). We now consider why the initial states are distributed in this manner.

The sol pressure $(p_{\textrm{f}}(t), p_{\textrm{r}}(t))$ first approaches a straight line $p_{\textrm{r}}=p_{\textrm{f}}+P_{\textrm{r}}^{\textrm{u}}-P_{\textrm{f}}^{\textrm{u}}$ in the absence of stimulation (Fig. 7(b)). The eigenvalues of Eq. (14) are negative, and the absolute value of the eigenvalue corresponding to the eigenvector $(1, -r_{\textrm{v}})^t$ is larger than that corresponding to the eigenvector $(1, 1)^t$ due to the diffusion effect (Eqs. (15) and (16)). Thus, the sol pressure decays faster toward $(1, -r_{\textrm{v}})^t$ on the $(p_{\textrm{f}}, p_{\textrm{r}})$ plane. In addition, the sol pressure transitions along the straight line $p_{\textrm{r}}=p_{\textrm{f}}+P_{\textrm{r}}^{\textrm{u}}-P_{\textrm{f}}^{\textrm{u}}$ after approaching this line, because Eq. (14) has the eigenvector $(1, 1)^t$ (Fig. 7(b)).

When crossing the b-Q, stimulation was absent during the last period of the day, but was present earlier in the day. Therefore, the sol pressure returns to a straight line $p_{\textrm{r}}=p_{\textrm{f}}+P_{\textrm{r}}^{\textrm{u}}-P_{\textrm{f}}^{\textrm{u}}$ although it leaves the line for some time when crossing the b-Q (Figs. 9(a) and 10(a)). When crossing the b-N, the sol pressure transitions along the straight line $p_{\textrm{r}}=p_{\textrm{f}}+P_{\textrm{r}}^{\textrm{u}}-P_{\textrm{f}}^{\textrm{u}}$ (Fig. 9(b)).

Consequently, the initial states are distributed along a straight line $p_{\textrm{r}}=p_{\textrm{f}}+P_{\textrm{r}}^{\textrm{u}}-P_{\textrm{f}}^{\textrm{u}}$ (Fig. 9). From this distribution, the sol pressure transitions along a straight line when crossing the b-N, independent of the bridge conditions of the previous day (Fig. 9(b)). Thus, the locomotion velocity remained constant (Fig. 6).

**Fig. 11:**
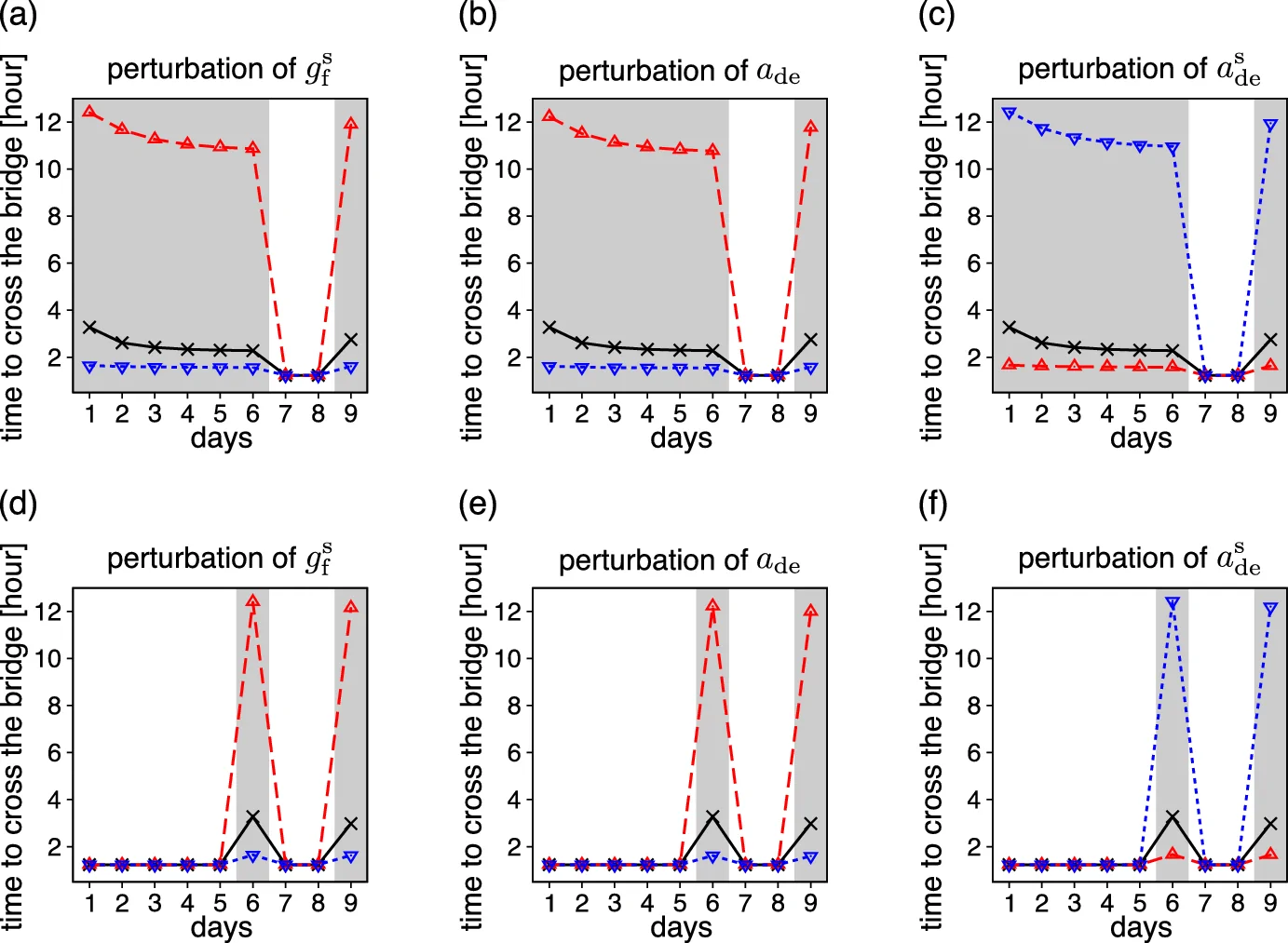
Time to cross the bridge corresponding to Figs. 5(a) and (b) when parameters $g_{\textrm{f}}^{\textrm{s}}$, $a_{\textrm{de}}$, and $a_{\textrm{de}}^{\textrm{s}}$ were individually perturbed by $\pm 5 \%$ from the values shown in the caption of Fig. 5. Black cross marks represent the original result corresponding to Figs. 5(a) and 5(b). Red upward triangle markers represent the result of a +5% perturbation applied to a parameter. Blue downward triangle markers represent the result of a -5% perturbation applied to a parameter. Gray and white backgrounds indicate the b-Q and b-N conditions, respectively. (a) Perturbation of $g_{\textrm{f}}^{\textrm{s}}$ for Fig. 5(a). (b) Perturbation of $a_{\textrm{de}}$ for Fig. 5(a). (c) Perturbation of $a_{\textrm{de}}^{\textrm{s}}$ for Fig. 5(a). (d) Perturbation of $g_{\textrm{f}}^{\textrm{s}}$ for Fig. 5(b). (e) Perturbation of $a_{\textrm{de}}$ for Fig. 5(b). (f) Perturbation of $a_{\textrm{de}}^{\textrm{s}}$ for Fig. 5(b)

## Analysis of parameter variations

Through parameter sensitivity analysis, we identified highly sensitive parameters, $g_{\textrm{f}}^{\textrm{s}}$, $a_{\textrm{de}}$, and $a_{\textrm{de}}^{\textrm{s}}$. In particular, the simulation results for the time to cross the bridge corresponding to Fig. 5(a) and (b) exhibited high sensitivity (see Online Resource 1). We then perturbed parameters $g_{\textrm{f}}^{\textrm{s}}$, $a_{\textrm{de}}$, and $a_{\textrm{de}}^{\textrm{s}}$ individually by $\pm 5\%$ and plotted the resulting bridge crossing times corresponding to Figs. 5(a) and (b) (Fig. 11). Note that, in some cases, the time to cross the b-Q exceeds $T_\textrm{s}=8$, which represents the stimulation duration (Fig. 11). Due to the perturbation, deceleration under stimulation becomes stronger, resulting in a negative velocity and causing the tip position ℓ to retreat. As a result, the model fails to complete crossing the bridge during stimulation and instead advances and crosses the bridge after the stimulation is removed. Consequently, cases in which the bridge crossing time exceeds $T_\textrm{s}=8$ do not necessarily reflect to the experimental setup, but rather represent mathematically computable outcomes of the model.

Even under parameter perturbations, the decline of time to cross the b-Q with repeated crossings was preserved. In addition, the recovery of time to cross the b-Q after passing through b-N also remained. These results indicate that the qualitative properties of the model output are maintained although quantitative changes are induced by the perturbations.

Time to cross the b-Q exhibits large variations under perturbation (Fig. 11). Since $g_{\textrm{f}}^{\textrm{s}}$ promotes the increase of $p_{\textrm{f}}$ (Eq. (4)), a larger $g_{\textrm{f}}^{\textrm{s}}$ enhances deceleration (Fig. 3(b)). Likewise, under stimulation, $a_{\textrm{de}}$, which corresponds to the degradation rate of $p_{\textrm{r}}$ (Eq. (5)), also promotes deceleration when increased (Fig. 3(b)). In contrast, $a_{\textrm{de}}^{\textrm{s}}$, representing the degradation rate of $p_{\textrm{f}}$ (Eq. (4)), enhances deceleration when decreased (Fig. 3(b)). Accordingly, a +5% perturbation in $g_{\textrm{f}}^{\textrm{s}}$ or $a_{\textrm{de}}$, as well as a -5% perturbation in $a_{\textrm{de}}^{\textrm{s}}$, results in an increase in the time to cross b-Q (Fig. 11).

No substantial variation is observed in time to cross the b-N. From Sect. 4.3, the velocity at b-N crossing is expressed as follows:

22 $$\begin{aligned} \dot{\ell }(t) \approx \gamma (P_{\textrm{r}}^{\textrm{u}}-P_{\textrm{f}}^{\textrm{u}})=\frac{g_{\textrm{r}}-g_{\textrm{f}}}{a_{\textrm{de}}+r_{\textrm{v}}d +d}. \end{aligned}$$

According to Eq. (22), the velocity at b-N crossing is independent of $g_{\textrm{f}}^{\textrm{s}}$ and $a_{\textrm{de}}^{\textrm{s}}$. Although $a_{\textrm{de}}$ appears in Eq. (22), its influence is limited because the value of *d* is sufficiently larger than $a_{\textrm{de}}$ ($a_{\textrm{de}}=2.244\times 10^{-2}$ and $d=4.909\times 10^{-1}$). Thus, a $\pm 5\%$ perturbation in $a_{\textrm{de}}$ does not significantly affect the velocity at b-N crossing. These considerations suggest that time to cross the b-N is insensitive to the perturbations.

## Discussion

### Core dynamics of habituation behavior in the proposed model

In the mathematical model proposed in this study, crawling migration (Eq. (1)) is regulated by the spatial difference in the protoplasmic pressure between the frontal and rear parts (Eqs. (2) and  (3)), and exhibits the two hallmarks defined in Table 1: #1 habituation and #2 spontaneous recovery.

The diffusion effect contributed to the transition of the initial state. Owing to diffusion effects, $p_{\textrm{r}}$ increases with increasing $p_{\textrm{f}}$ under stimulation. This, in turn, increases the displacement of $(p_{\textrm{f}}, p_{\textrm{r}})$ in the direction parallel to the iso-velocity lines (Fig. 9(a)). In the absence of stimulation, the sol pressure $(p_{\textrm{f}}, p_{\textrm{r}})$ returns to a straight line $p_{\textrm{r}}=p_{\textrm{f}}+P_{\textrm{r}}^{\textrm{u}}-P_{\textrm{f}}^{\textrm{u}}$ (Sect. 4.3). The combination of these behaviors with and without stimulation creates a 7-shaped trajectory on the phase plane (Fig. 10(a)), whereby an initial state shift along the straight line $p_{\textrm{r}}=p_{\textrm{f}}+P_{\textrm{r}}^{\textrm{u}}-P_{\textrm{f}}^{\textrm{u}}$ is realized.

The spatial difference in the chemical decomposition rate contributes to the connection between the initial state shift and mitigation of deceleration. The relationship between the decomposition rates under stimulation in the front and rear parts determines the slope of the iso-acceleration line Eq. (21). In our simulation, $a_{\textrm{de}}^{\textrm{s}}>a_{\textrm{de}}$ is true. Thus, the slope is greater than 1. However, the direction of the initial state shift is parallel to the iso-velocity line, whose slope is equal to 1 (Eq. (19)). Therefore, the direction of the initial state shift and the iso-velocity line intersect (Fig. 9(a)). Consequently, the deceleration was mitigated.

In summary, the diffusion effect between the front and rear parts is crucial for the transition of the initial state, and the difference in the chemical decomposition rate between the front and rear parts is important for reflecting the transition in the locomotion velocity. In short, the spatial scale of plasmodium played a key role in adaptive behavior in our model.

###  Comparison with the other models previously proposed

Some models exhibiting hallmarks #1 and #2 (Table 1) have been proposed (Eckert et al. 2024; Stanley 1976). In a model based on the biochemical kinetics of cellular movement, two distinct chemical species—activators and inhibitors—control the output (Eckert et al. 2024). In a model based on signal processing in neural circuits, the output is activated by the firing of other neurons and inhibited by a decline in the synaptic connection strength (Stanley 1976). In both models, the core mechanism controls the balance between two antagonistic factors: activation and inhibition of the response.

Although the biological realities represented by these models differ from those of our model, we considered whether there is any similarity in the core mechanism between our model and previously proposed models. In previous models, a common dynamic, which controls the balance between the inhibitory and activating effects on the response, was found.

In our model, the core mechanism lies in the relationship between the pressure difference of the protoplasm and the locomotion speed. In our simulation, the deceleration of locomotion speed under stimulation was decreased by repeated stimulation (Fig. 6). Thus, we regarded deceleration during stimulation as a response to the stimulation. The variable $p_{\textrm{f}}$ acts as an activator for deceleration under stimulation because a higher $p_{\textrm{f}}$ decreases locomotion speed (Eq. (1)). In contrast, the variable $p_{\textrm{r}}$ acts as an inhibitor of deceleration under stimulation because a higher $p_{\textrm{r}}$ increases locomotion speed (Eq. (1)). In this sense of a dynamical system, our model has a mechanism similar to that of previous models.

However, the novel point worth noting in our model is that the two variables of the activator and inhibitor are not different chemical components, but the same component in different places in plasmodium (Fig. 3). Spatial differences in identical biochemical processes can exhibit two hallmarks of habituation. In other words, our model suggested that the spatial inhomogeneity of a chemical plays a crucial role in the habituation of spatially extending cells, such as *P. polycephalum*.

**Fig. 12:**
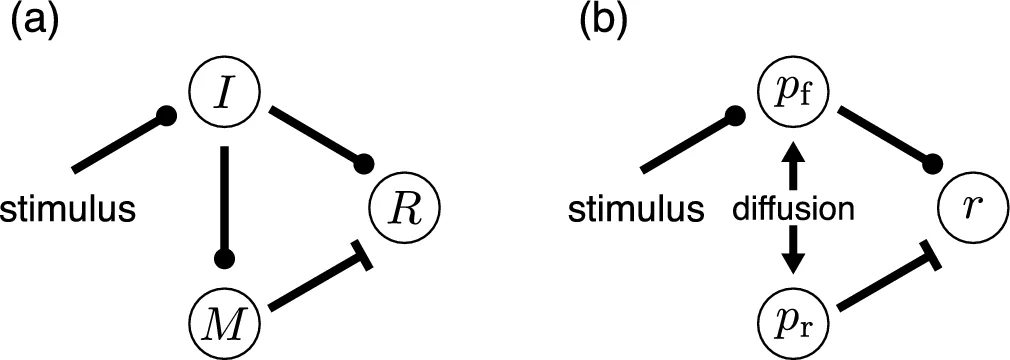
The schematic diagram of a model in Eckert et al. (2024) and our model. Line with a circle represents activation effect, line with a T-shaped end represents inhibition effect. (a) The schematic diagram of incoherent feed-forward (IFF) model in Eckert et al. (2024). *I* denotes input, *M* denotes memory, and *R* denotes response. (b) The schematic diagram of our model. *r* is equal to $-\dot{\ell }$. Double-headed arrow represents diffusion effect. Note that, due to differences in variable definitions, the effects of $p_{\textrm{f}}$ and $p_{\textrm{r}}$ on node *r* are reversed compared with their effects on node $\dot{\ell }$ in Fig. 3(b)

###  Biological differences and mathematical similarities between IFF model and our model

We specifically focus on the model proposed by Eckert et al. (2024) in the comparison. They developed a model based on concatenated incoherent feed-forward (CIFF) motifs, which describes a wider range of habituation hallmarks. A single incoherent feed-forward (IFF) motif can reproduce hallmarks #1 and #2 (Table 1) that our model does (Eckert 2022). In the IFF model, two factors, the input variable *I* and the memory variable *M*, act on the response *R* (Fig. 12(a)). *I* acts activatory on *R*, whereas *M* acts inhibitory on *R*. The activation effect under stimulation from *I* to *R* was constant for each stimulation. However, the inhibitory effect under stimulation from *M* to *R* depends on the series of stimulations. *M* increases during stimulation and decreases when stimulation is withheld. The IFF model realized hallmarks #1 and #2 (Table 1) from the above properties.

In our model, $p_{\textrm{f}}$ and $p_{\textrm{r}}$ affect the locomotion velocity $\dot{\ell }$. As previously mentioned, we defined deceleration under stimulation as the response to stimulation. To make it easier to compare the IFF model with ours, we regard an increase in $r(t)=-\dot{\ell }(t)$ as the response. $p_{\textrm{f}}$ is an activator of *r* and $p_{\textrm{r}}$ is its inhibitor (Fig. 12(b)). $p_{\textrm{f}}$ increased under stimulation. In addition, $p_{\textrm{r}}$ increased under stimulation because of the diffusion effect (Figs. 4(a) and 7(a)). For a more complex situation in which both the activator and inhibitor change, we analyzed the behavior of $(p_{\textrm{f}}(t), p_{\textrm{r}}(t))$ in the phase plane. The initial state shift was important for the decline and recovery of the response (Sect. 4.2 and  4.3). In our model, stimulation history was reflected in the initial state shift. To summarize, a common feature of the IFF model and our model is that they include a structure that stores the stimulation history. In the IFF model, variable *M*(*t*) plays the role of memory. In our model, this is the initial state transition in the phase plane.

CIFF model (Eckert et al. 2024) describes not only hallmarks #1 and #2, but also hallmarks #3–#6 and #10 (Table 1). Following a procedure similar to that used in Eckert et al. (2024), we verified hallmarks #3–#6 and #10 for the locomotion velocity in our model (Fig. 6), with the exception of #5, as our model does not account for changes in stimulation intensity (Appendix B.1). Hallmarks #3 and #6 were also observed in our model, although they were less pronounced than in the CIFF model. In contrast, hallmark #10 was not observed because the recovery time was short relative to the process of repeated stimulation. Hallmark #4 was also not observed in our model (Appendix B.2). In the CIFF model, these hallmarks are realized through the difference in timescales between the two memory variables (Eckert et al. 2024). Our model incorporates practically a single memory variable, because memory is represented by a shift along an iso-velocity line on the $(p_{\textrm{f}}, p_{\textrm{r}})$ plane (Fig. 5). In addition, whereas the CIFF model contains 14 model parameters, our model contains only 7 parameters. Therefore, our model is likely less capable of reproducing the hallmarks of habituation comprehensively than the CIFF model. Nevertheless, extending our model to include multiple timescales, as in Eckert et al. (2024), could capture broader hallmarks of habituation. Developing such a framework represents a promising direction for future research.

###  Biological background of our model

Responses to stimulus in *Physarum* plasmodium vary depending on body size and across different timescales. Therefore, we focus primarily on responses similar in size and timescale to those of the plasmodium used in the habituation experiments. The timescale is particularly important; we largely omit rapid responses occurring in ten minutes and instead refer mainly to slower responses emerging over several hours.

A back-and-forth protoplasmic flow occurs in approximately 2-minute cycles. But net transport is biased toward the front region and this bias is accumulated over many cycles in a longer timescale. Consequently, the leading edge thickens and advances. This protoplasmic flow is driven by the pressure difference generated between the front and rear regions. The pressure is generated by the active contraction of actomyosin. This contraction is regulated by chemical factors including ATP, $\text {Ca}^{2+}$, phospholipids, and cAMP (Ueda 1993). Differences in the concentrations of these chemical factors between the front and rear regions create disparities in contractile force and pressure, altering the speed (and direction) of migration. In our model, the concentrations of these chemical factors at the front and rear are reduced to single variables $c_{\textrm{f}}$ and $c_{\textrm{r}}$. In addition, contractile force and pressure at the front and rear are represented by $p_{\textrm{f}}$ and $p_{\textrm{r}}$, and the speed of migration is represented by $\dot{\ell }$.

When plasmodia migrate on agar gel over hours, the intracellular state differs between the front and rear of the plasmodium (Ueda et al. 1987, 1988). Changes in ATP concentration have been extensively studied regarding these speed variations on a long timescale over several hours. Intracellular ATP is not uniform throughout the cell; it is high in the front region and decreases gradually toward the rear. In typical eukaryotic cells, intracellular ATP is often maintained at a constant level by compensatory mechanisms like the creatine kinase. However, slime molds are known to lack this enzyme. High ATP concentrations soften the cell, suppressing contractile force generation (Ueda et al. 1987), allowing protoplasm pushed from the rear to the front. This propels the plasmodium forward.

When *V* decreases, the ATP concentration $[ATP]_{i}$ at the front region decreases, reducing the $[ATP]_{i}$ difference between the front and rear regions (Ueda et al. 1988). The migration speed *V* is related by $V\propto ([ATP]_{i}-[ATP]_{0})^{0.7}$(in the mM order). Here, since $[ATP]_{i}$ in the rear region is nearly at the threshold constant concentration $[ATP]_{0}$, the front-rear $[ATP]_{i}$ difference determines *V*. Comparing with experimental results, it is reasonable to approximate *V* as simply proportional to the front-rear $[ATP]_{i}$ difference.

It is known that intracellular chemical pattern formation (concentration gradients between front and rear) involving not only ATP but also $\text {Ca}^{2+}$, $\text {H}^{+}$, cAMP, phospholipids, etc., is involved in chemotaxis (Ueda 1993). What is certain is that chemical pattern formation controls this process. It is reported that biochemical states at the anterior and posterior regions are different even in normal-sized cells such as cellular slime molds (Iwamoto et al. 2025), fish keratocytes, leukocytes, and fibroblasts, widely observed in amoeboid movement (Bray 2001). Therefore, we formalize the intracellular difference between the anterior and posterior regions of the cell.

The process by which the chemicals in the agar gel inhibit the migration V involves two intracellular events: E1) responses in intracellular chemical mediators (ATP decreases) and, E2) pressure changes by modulation of actomyosin contraction. In our model, the migration V corresponds to the dynamics of $\dot{\ell }$, E1 corresponds to the dynamics of $c_{\textrm{f}}$ and $c_{\textrm{r}}$, and E2 corresponds to the dynamics of $p_{\textrm{f}}$ and $p_{\textrm{r}}$. From E2 to V, we assumed simple Hagen-Poiseuille flow (Eq. (1)). Because, while more detailed analyses have recently been actively pursued for flow networks (Kramar and Alim 2021), porous media (Radszuweit et al. 2014), and two-phase flow models (Guy et al. 2011), in all of these, flow rate is proportional to the stress gradient.

For the transition from E1 to E2, contraction force measurement results exist. However, the results are on the order of minutes, making it difficult (if not impossible) to directly relate them to changes occurring over hours. As described above, the linear relationships from E1 to V can be justified experimentally, and the linear relationship from E2 to V can be assumed by the theoretical considerations. Thus, we assumed a linear relationship from E1 to E2 as a sufficient condition to satisfy these linear relationships (Eq. (8)). As mentioned above, although we approximated the relationship from E1 and V as linear in this study, it is likely nonlinear. Accordingly, a nonlinear relationship from E1 to E2 would be expected. However, incorporating nonlinearity to develop a more physiologically plausible model remains a subject for future work.

### Future prospects for mathematical modeling of habituation behaviour

In the behavioral definition of habituation, there are ten hallmarks (Rankin et al. 2009). Some models have already been proposed to reproduce three or more hallmarks, but these models are more complicated than our model, as they involve more variables and incorporate nonlinearity in ordinary differential equations (ODEs) (Eckert et al. 2024; Smart et al. 2024), whereas our model essentially comprises two linear ODEs with switching parameters (Eqs. (2) and (3)). For example, Eckert et al. (2024) reproduced other hallmarks by concatenating two IFF models. Our model was constructed in a bottom-up approach and it has the potential to be improved by adding new variables and/or nonlinearities, thereby reproducing three or more hallmarks.

In this study, we developed a mathematical model to describe the two hallmarks, #1 habituation and #2 spontaneous recovery, in *P. polycephalum*. Specifically, we demonstrated that a specific chemical substance is distributed throughout its large body, and that the differences in chemical reactions between the front and rear regions allow for a description of these two hallmarks. Our analysis of this model revealed that the initial state transition on an iso-velocity line is important for the memory of stimulation history (Sect. 4.2 and  4.3). Our model provides an understanding of these two hallmarks from the perspective of a dynamic system. Our future research direction will be to analyze other hallmarks of habituation from the same perspective. This approach will advance our comprehensive understanding of habituation mechanisms among a wide range of species.

## Supplementary Information

Below is the link to the electronic supplementary material.

Supplementary file 1 (pdf 434 KB)

## Figures and Tables

**Fig. 13:**
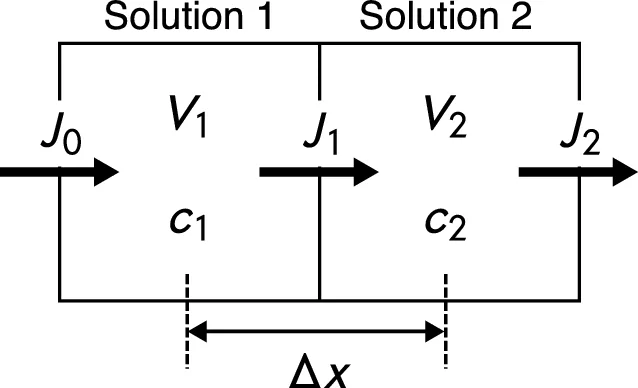
The problem setting of the diffusion between the different volumes of solutions. Arrow represents the flux

**Fig. 14:**
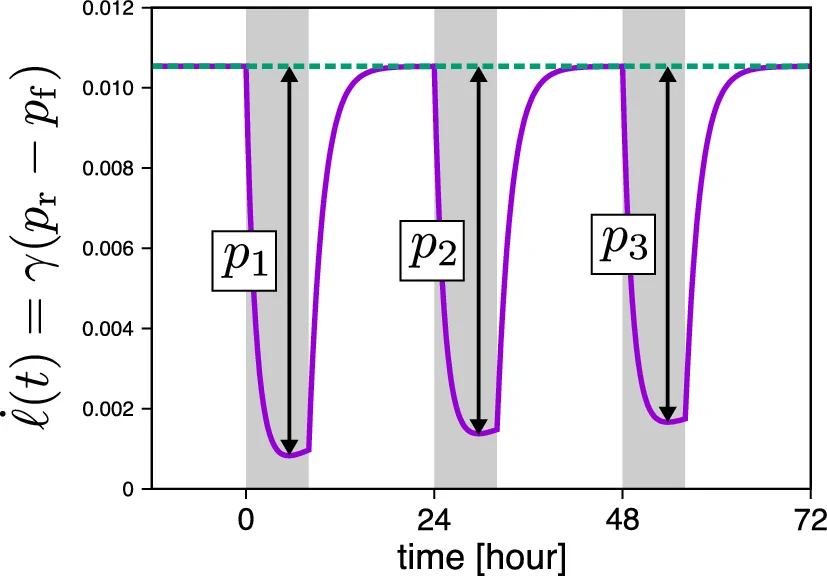
The description of $p_i$ in our model. Purple line represents *v*(*t*), green broken line represents that the velocity is equal to $v_\textrm{base}$, gray background represents the term under stimulation, h(t)=1, and white background represents the term in the absence of stimulation, h(t)=0

## Data Availability

The experimental data used in this study were originally generated by Boisseau et al. (2016) and are publicly available at https://doi.org/10.5061/dryad.51j89. The simulation codes are publicly available at https://doi.org/10.5281/zenodo.20438610.

## A Derivation of the Diffusion Term

We derived the diffusion term for different solution volumes. We consider adjacent solutions 1 and 2 (Fig. 13). The volume of solution *i* was defined as $V_i$ [$\hbox {m}^3$]. The chemical concentration of solution *i* is defined as $c_i$ [mol/$\hbox {m}^3$]. $J_i$ represents the flux [mol/$\hbox {m}^2$ · s]. We derived the diffusion term when $V_1 \ne V_2$. In this study, we assumed that the flux from both ends is zero; that is, $J_0=J_2=0$. From Fick’s first law, $J_1$ can be expressed as follows:

$$\begin{aligned} J_1=-D \frac{c_2-c_1}{\Delta x}, \end{aligned}$$

where *D* represents the diffusion coefficient [$\hbox {m}^2$/s], and Δx is the distance between solutions 1 and 2 [m]. Note that the flux $J_i$ is positive to the right. The inflow and outflow of solute particles per unit time in solution 1 are $S(J_0-J_1)$ and where *S* denotes the particle passage area [$\hbox {m}^2$]. Thus, the following equation is obtained:

23 $$\begin{aligned} \frac{d}{dt}(V_1 c_1)&= S(J_0-J_1)\nonumber \\&=\frac{SD}{\Delta x}(c_2 -c_1). \end{aligned}$$

From Eq. (23), the differential equation for $c_1$ is derived as

$$\begin{aligned} \frac{d}{dt}c_1(t)=\frac{SD}{V_1\Delta x}(c_2 -c_1). \end{aligned}$$

Similarly, the differential equation for $c_2$ is derived as follows:

$$\begin{aligned} \frac{d}{dt}c_2(t)=\frac{SD}{V_2\Delta x}(c_1 -c_2). \end{aligned}$$

We now define *d* and $r_{\textrm{v}}$ as follows:

$$\begin{aligned} d=\frac{SD}{V_2\Delta x}, r_{\textrm{v}}=\frac{V_2}{V_1}. \end{aligned}$$

Ultimately, we obtain the differential equation with the diffusion term between different volumes of the solutions as follows:

$$\begin{aligned} \frac{d}{dt}c_1(t)&=r_{\textrm{v}}d(c_2 -c_1), \\ \frac{d}{dt}c_2(t)&=d(c_1 -c_2). \end{aligned}$$

## B Verification of habituation hallmarks

### B.1 Method

Following Eckert et al. (2024), we conducted validations of hallmarks #3 potentiation of habituation, #4 frequency sensitivity, #6 subliminal accumulation, and #10 long-term habituation (Table 1). The trials to habituation $h_t\, [\textrm{stimuli}]$ was defined as the number of stimuli required for the relative change in the response to first fall below a threshold. We adopted a threshold of 1% following Eckert et al. (2024). Namely, $h_t = i$ when $(p_i-p_{i+1})/p_i<0.01$ for the first time, where $p_i$ denotes the peak height for the *i*th stimulation. In this study, we define the $p_i$ as follows:

$$\begin{aligned} p_{i}&= v_{\textrm{base}} - v(t_i), \\ v_{\textrm{base}}&= \gamma (P_{\textrm{r}}^{\textrm{u}}-P_{\textrm{f}}^{\textrm{u}}), \\ v(t_i)&= \gamma (p_{\textrm{r}}(t_i)-p_{\textrm{f}}(t_i)), \end{aligned}$$

where $t_i$ represents the time at which *v*(*t*) reaches its minimum in response to the *i*th stimulation (Fig. 14). The recovery time $r_t\, \text {[h]}$ was defined as the time required for the relative change between the initial response and the response to the test stimulation after habituation to fall below a threshold. We adopted a threshold of 5% following Eckert et al. (2024).

Hallmark #4 (frequency sensitivity) was evaluated by analyzing the changes in $h_t$ and $r_t$ in response to variations in the stimulation input period. Following Eckert et al. (2024), the stimulation input period was controlled by varying *T*, duration of one period, while keeping $T_\textrm{s}$, the stimulation duration, fixed. The definitions of *T* and $T_\textrm{s}$ are given in Sect. 3.3. Hallmark #3 (potentiation of habituation) was evaluated by first stopping the stimulation after habituation, withholding it for half of $r_t$, and then resuming the stimulation. The evaluation was based on the values of $h_t$ obtained in the first and second habituation process. Hallmark #6 (subliminal accumulation) was evaluated by continuously stimulating the system after habituation. Specifically, the stimulation was maintained until the relative change in output following two consecutive stimuli declined from the standard habituation threshold of 1% to 0.5%, after which the subsequent recovery time, $r_t$, was measured. Through this approach, we evaluated how the recovery time, $r_t$, changes in response to the application of additional stimulation after habituation. No explicit evaluation criterion was provided for hallmark #10 (long-term habituation). In CIFF model, the recovery time $r_t$ was sufficiently longer than the time required for the habituation process, and therefore hallmark #10 was treated in the same manner as hallmark #3 (Eckert et al. 2024). Therefore, we compared the recovery time $r_t$ with the time required for habituation $h_t T$ in our model.

**Table 2:** Hallmark #4 and #10

$T_\textrm{s}$ $\text {[h]}$	*T* $\text {[h]}$	$h_t$ $[\textrm{stimuli}]$	$r_t$ $\text {[h]}$	$h_t T$ $\text {[h]}$
8	32	2	13.64	64
8	24	3	31.80	72
8	16	5	53.84	90

### B.2 Results

As the period, *T*, was shortened, the number of stimulations required for habituation, $h_t$, increased, while the recovery time from habituation, $r_t$, became longer (Table 2). This behavior was opposite to hallmark #4 (Table 1), indicating that our model did not exhibit it. The time required for habituation, $h_t T$, was longer than the recovery time, $r_t$ (Table 2). Our model can only retain the stimulation history for a short duration. Consequently, hallmark #10 was not demonstrated.

The number of stimulations required for the second habituation process tended to be slightly smaller than that required for the first habituation process (Table 3). This is because, during the unstimulated period following the first habituation process, the pressures $(p_{\textrm{f}}, p_{\textrm{r}})$ do not fully return to their original state, similar to the behavior shown in Fig. 9(b). In addition, when additional stimulation was applied after habituation, the recovery time tended to become slightly longer (Table 4). This arises because the initial state shift of $(p_{\textrm{f}}, p_{\textrm{r}})$ is still induced even after the system has been habituated, similar to the behavior shown in Fig. 9(a). These behaviors correspond to hallmarks #3 and #6, respectively, suggesting that our model exhibited these properties, although only weakly.

**Table 3:** Hallmark #3

$T_\textrm{s}$ $\text {[h]}$	*T* $\text {[h]}$	$h_t$ (first) $[\textrm{stimuli}]$	$h_t$ (second) $[\textrm{stimuli}]$
8	32	2	1
8	24	3	2
8	16	5	4

**Table 4:** Hallmark #6

$T_\textrm{s}$ $\text {[h]}$	*T* $\text {[h]}$	$r_t$ (normal; $\textrm{threshold}=1\%$) $\text {[h]}$	$r_t$ (additional; $\textrm{threshold}=5\%$) $\text {[h]}$
8	32	13.64	19.29
8	24	31.80	35.32
8	16	53.84	56.96

## References

[CR1] Boisseau RP, Vogel D, Dussutour A (2016) Habituation in non-neural organisms: evidence from slime moulds. Proc Biol Sci 283(1829):20160446. 10.1098/rspb.2016.0446 27122563 PMC4855389

[CR2] Bray D (2001) Cell Movements: From Molecules to Motility, 2nd edn. Garland Publishing, New York, 10.4324/9780203833582

[CR3] Castellucci V, Pinsker H, Kupfermann I, Kandel ER (1970) Neuronal mechanisms of habituation and dishabituation of the gill-withdrawal reflex in Aplysia. Science 167(3926):1745–1748. 10.1126/science.167.3926.1745 5416543

[CR4] Dussutour A (2021) Learning in single cell organisms. Biochem Biophys Res Commun 564:92–102. 10.1016/j.bbrc.2021.02.018 33632547

[CR5] Eckert L (2022) Learning in single cells: Computational models of habituation. ETH Zurich, p 44 (**Master&apos;s Thesis**)

[CR6] Eckert L, Vidal-Saez MS, Zhao Z, Garcia-Ojalvo J, Martinez-Corral R, Gunawardena J (2024) Biochemically plausible models of habituation for single-cell learning. Curr Biol 34(24):5646-5658.e3. 10.1016/j.cub.2024.10.041 39566497

[CR7] Iwamoto K, Matsuoka S, Ueda M (2025) Excitable ras dynamics-based screens reveal rasgefx is required for macropinocytosis and random cell migration. Nat Commun 16(1):117. 10.1038/s41467-024-55389-2 39746985 PMC11696275

[CR8] Johnson MC, Wuensch KL (1994) An investigation of habituation in the jellyfish Aurelia aurita. Behav Neural Biol 61(1):54–59. 10.1016/S0163-1047(05)80044-5 8129686

[CR9] Guy RD, Nakagaki T, Wright GB (2011) Flow-induced channel formation in the cytoplasm of motile cells. Phys Rev E 84:016310. 10.1103/PhysRevE.84.016310 21867307

[CR10] Kramar M, Alim K (2021) Encoding memory in tube diameter hierarchy of living flow network. Proc Natl Acad Sci U S A 118(10):e2007815118. 10.1073/pnas.2007815118 33619174 PMC7958412

[CR11] Lyon P, Keijzer F, Arendt D, Levin M (2021) Reframing cognition: getting down to biological basics. Philos Trans R Soc Lond B Biol Sci 376(1820):20190750. 10.1098/rstb.2019.0750 33487107 PMC7935032

[CR12] Ma Q, Johansson A, Tero A, Nakagaki T, Sumpter DJT (2013) Current-reinforced random walks for constructing transport networks. J R Soc Interface 10(80):20120864. 10.1098/rsif.2012.0864 23269849 PMC3565737

[CR13] Nakagaki T, Yamada H, Tóth A (2000) Maze-solving by an amoeboid organism. Nature 407(6803):470–470. 10.1038/35035159 11028990

[CR14] Nakagaki T, Iima M, Ueda T, Nishiura Y, Saigusa T, Tero A, Kobayashi R, Showalter K (2007) Minimum-risk path finding by an adaptive amoebal network. Phys Rev Lett 99(6). 10.1103/PhysRevLett.99.068104 17930872

[CR15] Radszuweit M, Engel H, Bär M (2014) An active poroelastic model for mechanochemical patterns in protoplasmic droplets of Physarum polycephalum. PLoS ONE 9(6):e99220. 10.1371/journal.pone.0099220 24927427 PMC4057197

[CR16] Rajan D, Makushok T, Kalish A, Acuna L, Bonville A, Correa Almanza KC, Garibay B, Tang E, Voss M, Lin A, Barlow K, Harrigan P, Slabodnick MM, Marshall WF (2023) Single-cell analysis of habituation in Stentor coeruleus. Curr Biol 33(2):241-251.e4. 10.1016/j.cub.2022.11.010 36435177 PMC9877177

[CR17] Rankin CH, Abrams T, Barry RJ, Bhatnagar S, Clayton DF, Colombo J, Coppola G, Geyer MA, Glanzman DL, Marsland S, McSweeney FK, Wilson DA, Wu CF, Thompson RF (2009) Habituation revisited: an updated and revised description of the behavioral characteristics of habituation. Neurobiol Learn Mem 92(2):135–138. 10.1016/j.nlm.2008.09.012 18854219 PMC2754195

[CR18] Reid CR (2023) Thoughts from the forest floor: a review of cognition in the slime mould physarum polycephalum. Anim Cogn 26(6):1783–1797. 10.1007/s10071-023-01782-1 37166523 PMC10770251

[CR19] Saigusa T, Tero A, Nakagaki T, Kuramoto Y (2008) Amoebae anticipate periodic events. Phys Rev Lett 100(1):018101. 10.1103/PhysRevLett.100.018101 18232821

[CR20] Smart M, Shvartsman SY, Mönnigmann M (2024) Minimal motifs for habituating systems. Proc Natl Acad Sci U S A 121(41):e2409330121. 10.1073/pnas.2409330121 39365818 PMC11474051

[CR21] Stanley JC (1976) Computer simulation of a model of habituation. Nature 261(5556):146–148. 10.1038/261146a0 179015

[CR22] Takagi S, Nishiura Y, Nakagaki T, Ueda T, Ueda KI (2007) Indecisive behavior of amoeba crossing an environmental barrier. In: Topological aspects of critical systems and networks. World Scientific, pp 86–93, 10.1142/9789812708687_0011

[CR23] Tero A, Kobayashi R, Nakagaki T (2007) A mathematical model for adaptive transport network in path finding by true slime mold. J Theor Biol 244(4):553–564. 10.1016/j.jtbi.2006.07.015 17069858

[CR24] Tero A, Takagi S, Saigusa T, Ito K, Bebber DP, Fricker MD, Yumiki K, Kobayashi R, Nakagaki T (2010) Rules for biologically inspired adaptive network design. Science 327(5964):439–442. 10.1126/science.1177894 20093467

[CR25] Ueda T (1993) Pattern dynamics in cellular perception. Phase Transit 45(2–3):93–104. 10.1080/01411599308223719

[CR26] Ueda T, Mori Y, Kobatake Y (1987) Patterns in the distribution of intracellular ATP concentration in relation to coordination of amoeboid cell behavior in Physarum polycephalum. Exp Cell Res 169(1):191–201. 10.1016/0014-4827(87)90237-0 3817013

[CR27] Ueda T, Nakagaki T, Kobatake Y (1988) Patterns in intracellular ATP distribution and rhythmic contraction in relation to amoeboid locomotion in the plasmodium of *Physarum polycephalum*. In: Cell Dynamics: Cytoplasmic Streaming Cell Movement-Contraction and Migration Cell and Organelle Division Phototaxis of Cell and Cell Organelle. Springer, p 51–56, 10.1007/978-3-7091-9008-1_7

[CR28] Wood DC (1988) Habituation in Stentor: a response-dependent process. J Neurosci 8(7):2248–2253. 10.1523/JNEUROSCI.08-07-02248.1988 3249222 PMC6569516

[CR29] Wood DC (1988) Habituation in Stentor: produced by mechanoreceptor channel modification. J Neurosci 8(7):2254–2258. 10.1523/JNEUROSCI.08-07-02254.1988 3249223 PMC6569508

