# Supplementary material for: Mathematical modeling for a primitive form of habituation in an amoeba
Source: J Math Biol. 2026 Aug 17;93(3):36. doi: 10.1007/s00285-026-02438-7 (PMC13481565; doi:10.1007/s00285-026-02438-7)
Supplement: Supplementary file 1 [file 285_2026_2438_MOESM1_ESM.pdf]

Supplementary Information: Mathematical Modeling for a Primitive Form of Habituation in an Amoeba

Kota Nishi, Atsushi Tero, Yukinori Nishigami, Toshiyuki Nakagaki

S1 Parameter estimation

S1.1 Method

To identify the optimal parameters for our model, we performed parameter estimation by a global search and subsequently a local refinement. To efficiently explore the parameter space across several orders of magnitude, each parameter θ_i was optimized in a normalized $\log_{10}$-transformed space defined as

$$z_i = \frac{\log_{10}\theta_i - \log_{10}\theta_i^{\mathrm{low}}}{\log_{10}\theta_i^{\mathrm{high}} - \log_{10}\theta_i^{\mathrm{low}}},$$

where θ_i^{low}, θ_i^{high} represent the lower and upper bounds of the parameter θ_i, respectively. We implemented the optimization processes by Python 3.10.12.

First, a Genetic Algorithm (GA) was utilized for global exploration. The GA was implemented using the DEAP framework version 1.4.3 [1]. Details are provided in Table S1. We performed the GA optimization across 400 independent trials, using distinct seed values ranging from 0 to 399. In each trial, the population size was set to 200, and the evolution was continued for $10,000$ generations.

Subsequently, for each of the 400 GA trials, the top 20 parameter sets with the smallest loss function values were selected and used as initial values for local refinement. In this stage, the Nelder–Mead simplex algorithm was performed via the SciPy library version 1.15.3 [2]. Among the locally refined parameter sets obtained from each trial, the parameter set yielding the minimum loss function value was selected as the final estimate for that trial. As a result, 400 parameter sets corresponding to local minima were obtained.

The loss function was defined as follows:

$$F(\boldsymbol{\theta}) = \sum_{i\in\{\mathrm{Q,C1,C2}\}} \sum_{j=1}^{D} \frac{1}{N_i} \sum_{k=1}^{N_i} (T_j^i(\boldsymbol{\theta}) - T_{jk}^i)^2.$$

$T_j^i(\boldsymbol{\theta})$ and T_{jk}^i represent the time to cross the bridge from our model simulation and experimental results [3], respectively. The index i denotes the experimental condition. The condition Q, C1, and C2 represent nine-day experimental sequences, each characterized by distinct schedules of agar bridge presentation (Table S2). The index j represents day, and the index k denotes the each individual. N_i $(i \in \{\mathrm{Q,C1,C2}\})$ represent the number of individuals for each condition, and D represents the total days of experiment ($N_{\mathrm{Q}} = 68$, $N_{\mathrm{C1}} = 82$, $N_{\mathrm{C2}} = 100$, $D = 9$).

In our model, there are ten parameters, L_{r}, T_{s}, g_{f}, g_{r}, $g_{\mathrm{f}}^{\mathrm{s}}$, a_{de}, $a_{\mathrm{de}}^{\mathrm{s}}$, d, γ, and r_{v}. We fixed some parameters, L_{r}, T_{s}, and g_{r}. The parameter L_{r}, which denotes the bridge length, was fixed based on experimental setting, L_{r}=0.013 [3]. The parameter T_{s}, representing the duration of stimulus detection, was fixed to a value sufficiently exceeding time to cross the bridge, $T_{\mathrm{s}} = 8$, as its true value remains unknown. The parameter g_{r}, which is associated with the chemical generation rate, was fixed to $g_{\mathrm{r}} = 1$ through non-dimensionalization of our model. In summary, we fixed three parameters per ten parameters. The parameter set of loss function is defined in a seven-dimensional space, denoted as $\boldsymbol{\theta} \in \mathbb{R}^7$. The parameter set $\boldsymbol{\theta}$ is defined as follows:

$$(\theta_1, \theta_2, \theta_3, \theta_4, \theta_5, \theta_6, \theta_7)^t = (g_{\mathrm{f}}, g_{\mathrm{f}}^{\mathrm{s}}, a_{\mathrm{de}}, a_{\mathrm{de}}^{\mathrm{s}}, d, \gamma, r_{\mathrm{v}})^t.$$

S1.2 Results

We conducted parameter estimation within the parameter ranges listed in Table S3. We assumed that the front part is smaller than the rear part. Thus, the upper bound of r_{v} was set to 1. We confirmed the convergence of GA processes (Fig. S1). The top 5 results of parameter estimation are shown in Table S4. The

parameter g_f of several seeds reached the lower bound of the interval. Thus, we performed a re-estimation by extending the lower limit of g_f to 10^{-4}. However, g_f also reached the lower bound, and the loss function values increase slightly (data not shown). In addition, our model shows low sensitivity to the parameter g_f (Fig. S2; details are provided in Sec. S2). From these reasons, we did not expand the lower bound, and adopted the range in Table S3.

S2 Parameter sensitivity analysis

S2.1 Method

To identify the key parameters and examine the robustness of our model, we investigated the effects of parameter variations on the model output, specifically the time to cross the bridge, using a local sensitivity approach. A local sensitivity analysis was conducted by regarding the time to cross the bridge as a function of the parameters, g_f, g_f^s, a_de, a_de^s, d, γ, and r_v. The bridge crossing time can be expressed as follows:

$$T_k^i(\boldsymbol{\theta}), i \in \{\mathrm{Q}, \mathrm{C1}, \mathrm{C2}\}, k \in \{1, 2, \ldots, 9\}.$$

Here, index i denotes the experimental condition (Table S2). Index k denotes the day. Namely, $T_k^i(\boldsymbol{\theta})$ means the time to cross the bridge at day k under condition i. We defined the parameter sensitivity for each condition as follows:

$$S_j^i(\boldsymbol{\theta}^*) = \frac{1}{D}\sum_{k=1}^{D}\left|\frac{\theta_j^*}{T_k^i(\boldsymbol{\theta}^*)}\frac{\partial T_k^i}{\partial \theta_j}(\boldsymbol{\theta}^*)\right|,\ i \in \{\mathrm{Q}, \mathrm{C1}, \mathrm{C2}\}, j \in \{1, 2, \ldots, 7\},$$

where $\boldsymbol{\theta}^*$ represents a given parameter set, and D represents the total days of experiment (here, $D = 9$). We numerically approximated the differentiation as follows:

$$\frac{\partial T_k^i}{\partial \theta_j}(\boldsymbol{\theta}^*) \approx \frac{T_k^i(\theta_1^*, \ldots, \theta_j^* + \varepsilon\theta_j^*, \ldots, \theta_7^*) - T_k^i(\theta_1^*, \ldots, \theta_j^* - \varepsilon\theta_j^*, \ldots, \theta_7^*)}{2\varepsilon\theta_j^*}.$$

S2.2 Results

We conducted the parameter sensitivity analysis with $\varepsilon = 0.01$, using the best parameter set obtained from the parameter estimation as $\boldsymbol{\theta}^*$ (Seed 308; Table S4). The results revealed that the parameters g_f^s, a_de, and a_de^s exhibit high sensitivity, particularly under conditions Q and C1. Similar results were observed for the other parameter sets listed in Table S3 (data not shown).

References

[1] Fortin FA, De Rainville FM, Gardner MAG, et al (2012) Deap: Evolutionary algorithms made easy. The Journal of Machine Learning Research 13(1):2171–2175

[2] Virtanen P, Gommers R, Oliphant TE, et al (2020) Scipy 1.0: fundamental algorithms for scientific computing in python. Nature methods 17(3):261–272

[3] Boisseau RP, Vogel D, Dussutour A (2016) Habituation in non-neural organisms: evidence from slime moulds. Proc Biol Sci 283(1829):20160446.

[4] Bäck T (2018) Evolutionary computation 1: Basic algorithms and operators. CRC press

Table S1: The settings of Genetic Algorithm

Component	Description	DEAP function
Algorithm	Evolutionary algorithm in [4]	`algorithms.eaSimple(cxpb=0.5, mutpb=0.3)`
Individual	Vector of model parameters (Real-valued vector)	`creator.create("Individual", ...)`
Initialization	Uniform random distribution	`tools.initCycle`, `tools.initRepeat`
Selection	Tournament selection	`tools.selTournament(tournsize=2)`
Crossover	Blend crossover (BLX-alpha)	`tools.cxBlend(alpha=0.5)`
Mutation	Gaussian mutation	`tools.mutGaussian(mu=0, sigma=0.04, indpb=0.2)`

Table S2: The experimental conditions

Condition	Presentation schedule
Q	b-Q (Days 1–6, 9), b-N (Days 7–8)
C1	b-Q (Days 6, 9), b-N (Days 1–5, 7–8)
C2	b-N (Days 1–9)

[1] b-Q represents an agar bridge containing quinine, and b-N represents a normal agar bridge without quinine.

Table S3: Parameter ranges used for parameter estimation

parameter	range
g_{f}, $g_{\mathrm{f}}^{\mathrm{s}}$, a_{de}, $a_{\mathrm{de}}^{\mathrm{s}}$, d, γ	$[10^{-3}, 10^{2}]$
r_{v}	$[10^{-3}, 1]$

Table S4: Top 5 results of parameter estimation

Seed	Loss	g_{f}	$g_{\mathrm{f}}^{\mathrm{s}}$	a_{de}	$a_{\mathrm{de}}^{\mathrm{s}}$	d	γ	r_{v}
308	22.23951	1.001.E-03	1.197.E+01	2.244.E-02	2.866.E-01	4.909.E-01	5.782.E-03	7.088.E-02
300	22.23952	1.000.E-03	1.861.E+01	2.187.E-02	4.320.E-01	4.539.E-01	5.301.E-03	5.912.E-02
319	22.23971	1.074.E-03	1.393.E+01	2.263.E-02	3.343.E-01	4.403.E-01	5.181.E-03	6.422.E-02
12	22.23978	6.959.E-03	1.618.E+01	2.258.E-02	3.867.E-01	4.100.E-01	4.853.E-03	6.072.E-02
3	22.24002	1.187.E-02	1.586.E+01	2.129.E-02	3.623.E-01	6.349.E-01	7.457.E-03	6.796.E-02

[1] Loss function values are rounded to the fifth decimal place.
[2] Parameter values are expressed to four significant figures.

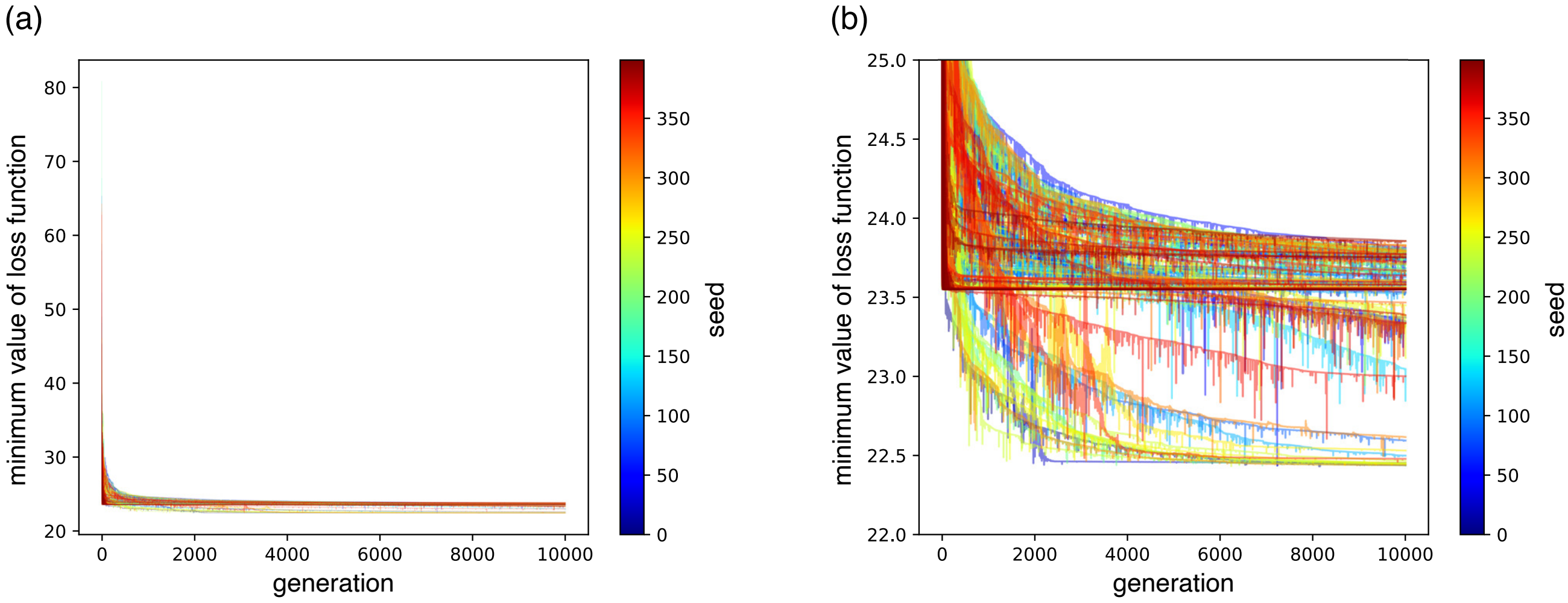

Figure S1: (a) Evolution of the loss function for each seed during the GA process. (b) Enlarged view of (a).

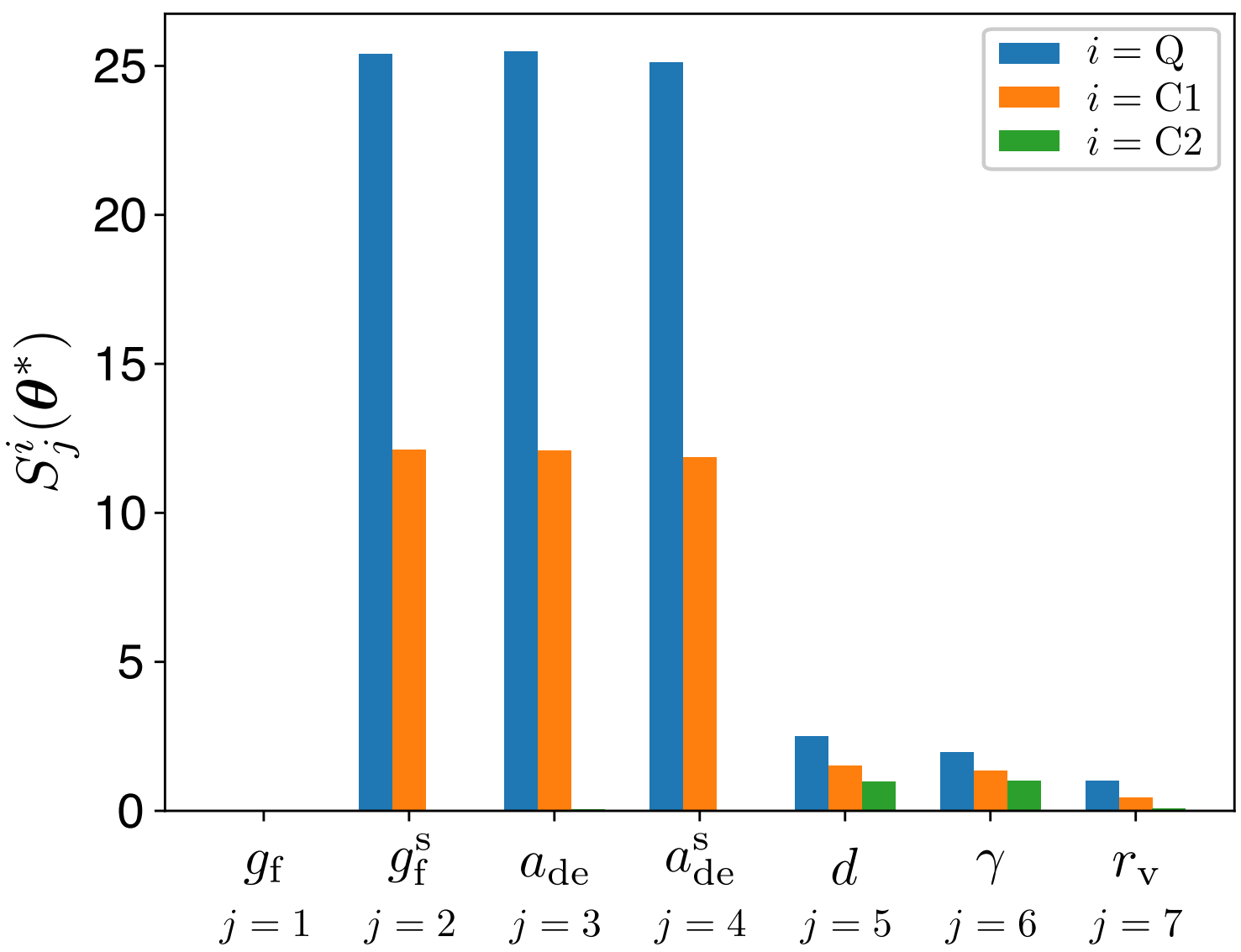

Figure S2: The result of the parameter sensitivity analysis for best parameter set (Seed 308; Table S4)
